# Inflammation and vascular remodeling in COVID-19 hearts

**DOI:** 10.1007/s10456-022-09860-7

**Published:** 2022-11-12

**Authors:** Christopher Werlein, Maximilian Ackermann, Helge Stark, Harshit R. Shah, Alexandar Tzankov, Jasmin Dinonne Haslbauer, Saskia von Stillfried, Roman David Bülow, Ali El-Armouche, Stephan Kuenzel, Jan Lukas Robertus, Marius Reichardt, Axel Haverich, Anne Höfer, Lavinia Neubert, Edith Plucinski, Peter Braubach, Stijn Verleden, Tim Salditt, Nikolaus Marx, Tobias Welte, Johann Bauersachs, Hans-Heinrich Kreipe, Steven J. Mentzer, Peter Boor, Stephen M. Black, Florian Länger, Mark Kuehnel, Danny Jonigk

**Affiliations:** 1 Institute of Pathology, Hannover Medical School, Carl-Neuberg-Straße 1, 30625 Hannover, Germany; 2 Institute of Pathology and Department of Molecular Pathology, Helios University Clinic Wuppertal, University of Witten/Herdecke, Wuppertal, Germany; 3 Institute of Functional and Clinical Anatomy, University Medical Center of the Johannes Gutenberg-University Mainz, Mainz, Germany; 4 Member of the German Center for Lung Research (DZL), Biomedical Research in Endstage and Obstructive Lung Disease Hannover (BREATH), Hannover, Germany; 5 Institute of Medical Genetics and Pathology, University Hospital Basel, Basel, Switzerland; 6 Institute of Pathology, RWTH University of Aachen, Aachen, Germany; 7 Institute of Pharmacology and Toxicology, Faculty of Medicine Carl Gustav Carus, Technische Universität Dresden, Dresden, Germany; 8 Department of Dermatology, Faculty of Medicine Carl Gustav Carus, Technische Universität Dresden, Dresden, Germany; 9 Department of Histopathology, Royal Brompton and Harefield NHS Foundation Trust, London, UK; 10 Institute for X-Ray Physics, University of Göttingen, Göttingen, Germany; 11 Department of Cardiothoracic, Transplantation and Vascular Surgery, Hannover Medical School, Hannover, Germany; 12 Department of Thoracic Medicine, Antwerp University Hospital, Antwerp, Belgium; 13 Cluster of Excellence ’Multiscale Bioimaging: From Molecular Machines to Networks of Excitable Cells’ (MBExC), University of Göttingen, Göttingen, Germany; 14 Department of Internal Medicine I, University Hospital Aachen, Aachen, Germany; 15 Clinic of Pneumology, Hannover Medical School, Hannover, Germany; 16 Department of Cardiology and Angiology, Hannover Medical School, Hannover, Germany; 17 Laboratory of Adaptive and Regenerative Biology, Brigham and Women’s Hospital, Harvard Medical School, Boston, USA; 18 Division of Thoracic Surgery, Brigham and Women’s Hospital, Harvard Medical School, Boston, USA; 19 Institute of Pathology and Department of Nephrology, RWTH University of Aachen, Aachen, Germany; 20 Department of Cellular Biology and Pharmacology Translational Medicine, Florida International University, Florida, USA

**Keywords:** COVID-19, Heart, Macrophages, Angiogenesis, Intussusception, Coronavirus disease 2019, Acute heart failure, Intussusceptive angiogenesis, CD11b, TIE2

## Abstract

A wide range of cardiac symptoms have been observed in COVID-19 patients, often significantly influencing the clinical outcome. While the pathophysiology of pulmonary COVID-19 manifestation has been substantially unraveled, the underlying pathomechanisms of cardiac involvement in COVID-19 are largely unknown. In this multicentre study, we performed a comprehensive analysis of heart samples from 24 autopsies with confirmed SARS-CoV-2 infection and compared them to samples of age-matched Influenza H1N1 A (*n* = 16), lymphocytic non-influenza myocarditis cases (*n* = 8), and non-inflamed heart tissue (*n* = 9). We employed conventional histopathology, multiplexed immunohistochemistry (MPX), microvascular corrosion casting, scanning electron microscopy, X-ray phase-contrast tomography using synchrotron radiation, and direct multiplexed measurements of gene expression, to assess morphological and molecular changes holistically. Based on histopathology, none of the COVID-19 samples fulfilled the established diagnostic criteria of viral myocarditis. However, quantification via MPX showed a significant increase in perivascular CD11b/TIE2 + —macrophages in COVID-19 over time, which was not observed in influenza or non-SARS-CoV-2 viral myocarditis patients. Ultrastructurally, a significant increase in intussusceptive angiogenesis as well as multifocal thrombi, inapparent in conventional morphological analysis, could be demonstrated. In line with this, on a molecular level, COVID-19 hearts displayed a distinct expression pattern of genes primarily coding for factors involved in angiogenesis and epithelial-mesenchymal transition (EMT), changes not seen in any of the other patient groups. We conclude that cardiac involvement in COVID-19 is an angiocentric macrophage-driven inflammatory process, distinct from classical anti-viral inflammatory responses, and substantially underappreciated by conventional histopathologic analysis. For the first time, we have observed intussusceptive angiogenesis in cardiac tissue, which we previously identified as the linchpin of vascular remodeling in COVID-19 pneumonia, as a pathognomic sign in affected hearts. Moreover, we identified CD11b + /TIE2 + macrophages as the drivers of intussusceptive angiogenesis and set forward a putative model for the molecular regulation of vascular alterations.

**Supplementary Information:**

The online version contains supplementary material available at 10.1007/s10456-022-09860-7.

## Clinical perspective

- Cardiac manifestations of COVID-19 represent a significantly underappreciated injury pattern in conventionally assessed specimens.
- As in other organs, cardiac involvement by COVID-19 is an angiocentric process, which in the heart appears to be primarily driven by macrophages.
- Vascular remodeling in the shape of intussusceptive angiogenesis with accompanying microthrombi may explain the observed clinical findings, such as arrhythmia, elevated troponin levels, and reduced ejection fraction.

## Introduction

Severe acute respiratory syndrome coronavirus 2 (SARS-CoV-2) primarily affects the respiratory system and may also evolve into a multi-organ disease [1], with virus-triggered vasculopathy, especially endothelial dysfunction, hypercoaguability, and microthrombi [2, 3] as a common denominator [4, 5] and predictor for disease severity [6–8]. Among the plethora of symptoms associated with coronavirus disease 2019 (COVID-19), 20–30% of hospitalized patients show cardiac function impairment with a myocarditis-like presentation [9]. They may suffer from arrhythmia, elevated troponin levels, lower ejection fraction, and ventricle wall dyskinesia [10]. Additionally, right ventricular dysfunction, in part due to increased afterload by pulmonary vascular involvement (e.g., microthrombi and vascular remodeling), is a common finding contributing to increased cardiac serum markers and acts as an independent risk factor for a severe cause of disease and mortality [11–13].

While an increasing number of COVID-19 cases with cardiac involvement are being reported, and the possibility of COVID-19-triggered chronic cardiac disease is under discussion, the actual pathomechanisms and sequence of heart injury in the affected patients remain unclear. Similar frequencies of cardiac symptoms in COVID-19 and SARS beta-coronavirus patients suggest a form of heart injury with myocyte damage and residual fibrosis comparable to other forms of viral myocarditis [14]. Unraveling the exact injury mechanism is complicated by the potential impact of preexisting cardiac conditions [15]. Also, cardiac injury, as defined by elevated serum markers such as troponins, is a rather common finding in patients in need of intensive care, regardless of the underlying disease [16].

Current hypotheses regarding cardiac injury in COVID-19 infection include both direct and indirect injury to the myocardium by viral infection. Cardiac COVID-19 infection has been described in a series of case reports describing clinical (peri-) myocarditis [17]. Thus, SARS-CoV-2 RNA has been reported anecdotally in cardiac tissue and two case reports described virus-shaped particles in cardiac macrophages [18] and cardiomyocytes [19]. Another hypothesis implicates the systemic release of pro-inflammatory cytokines (e.g., IL-1, IL-6, TNF-α, IFN-γ, and MIP) and the subsequent “cytokine storm” as the main culprit with subsequent increased vascular wall permeability and myocardial edema [20].

Conventional histopathology studies report mixed findings in COVID-19 hearts ranging from typical lymphocytic myocarditis, thrombotic microangiopathy to a lack of significant lymphocytic infiltrates and myocyte damage, with the majority of cases not fulfilling the established Dallas criteria for myocarditis [21]. The most frequently reported morphological finding has been a slight increase in perivascular macrophages, often referred to as borderline myocarditis [19, 22]. Interpreting the results is further hampered by the inconsistency of the used nomenclature regarding the terms myocarditis, inflammation, and cardiac involvement. Here, we decided to define myocarditis in line with the established Dallas criteria as conventional lymphocyte-driven inflammation, compared to macrophage-driven inflammatory reactions in COVID-19 and influenza hearts.

In order to provide a better understanding of the (ultra)structural changes and inflammatory microenvironment in the heart of COVID-19 patients, we employed a multimodal and comprehensive analysis of cardiac autopsy tissue via histology, immunohistochemistry, vascular corrosion casts, electron microscopy, and gene expression analysis using influenza (H1N1) cases, classic viral myocarditis (e.g., coxsackievirus), and non-infected control cases.

## Methods

### Patient selection and workflow

We analyzed cardiac autopsy specimens from 24 patients, who died from respiratory failure caused by SARS-CoV-2 infection and compared them with heart samples from 16 patients, who died from pneumonia caused by influenza A virus subtype H1N1 (A[H1N1])—a strain associated with the 1918 and 2009 influenza pandemics. The influenza samples were selected from archived tissue from Hannover Medical School and RWTH Aachen mainly from the 2009 pandemic as well as from 2010, 2011, and 2018, and chosen for the best possible match regarding age, sex, and disease severity. Eight heart samples of non-influenza and non-SARS-CoV-2 myocarditis were selected from archived tissue from routine diagnostics at Hannover Medical School and autopsy cases from university clinic Augsburg. Nine tissue samples from cardiac surgery other than infectious/inflammatory disease served as uninfected control specimens. The experiments performed were approved by the local ethics committee at Hannover Medical School (ethics vote number: 2893-2015 and 9022 BO K 2020). The COVID-19 group consisted of hearts from nine female and fifteen male patients with mean (± SD) ages of 76 ± 6.3 years and 72 ± 12.8 years, respectively. The influenza group consisted of hearts from seven female and nine male patients with mean (± SD) ages of 53 ± 16.7 years and 51 ± 15.2 years, respectively. The non-influenza and non-SARS-CoV-2 myocarditis group consisted of samples from three female and five male patients with mean (± SD) ages of 59 ± 29.5 years and 52 ± 18.4 years, respectively. The control group consisted of hearts from seven female and two male patients with mean (± SD) ages of 53 ± 20.1 years and 62.5 ± 3.5 years, respectively (patient characteristics and clinical data are provided in supplementary Tables 1 and 2).

Clinical data on cardiac involvement including serum parameters such as CK-MB, troponin, NT-proBNP, and echocardiography data were available in 16 of the 24 analyzed samples in the COVID-19 cohort, 12 of the 16 influenza cases, and 3 of the 8 non-influenza myocarditis cases, respectively. Clinical signs of cardiac involvement were defined by elevated serum markers, e.g., CK-MB, troponin, NT-proBNP, and/or abnormalities in echocardiography, e.g., reduced LVEF/RVEF or dyskinesia, and were found in 11 of the 16 surveyed COVID-19 cases, 9 of the 12 explored influenza cases and all 3 of the myocarditis cases (for full clinical data see supplementary Tables 1 and 2).

Heart samples were comprehensively analyzed, employing the whole spectrum of conventional histopathology, immunohistochemistry, multiplex immunohistochemistry, microvascular corrosion casting, synchrotron radiation tomographic microscopy (SRXTM), and gene expression analysis via the ncounter nanostring system. SARS-CoV-2 detection was performed by immunohistochemistry against SARS-CoV-2 spike and nucleocapsid protein, and the presence of RNA was detected using an RNA-FISH probe and RT-PCR after RNA isolation of FFPE samples. Specifically, from the available formalin-fixed unembedded tissue, 13 COVID-19 samples, 3 lymphocytic myocarditis samples, and 3 control samples were suitable for vascular corrosion casting and 3D scanning electron microscopy, the gold standard for detailed analysis of tissue the microvasculature, angiogenesis, and vascular occlusions, e.g., by microthrombi [4]. Additionally, FFPE paraffin blocks from 11 COVID-19 and 5 control heart samples were investigated by phase-contrast X-ray tomography at the GINIX endstation of the P10 beamline at the PETRAIII storage ring 104 (DESY, Hamburg), a novel technique for assessing especially the capillary network, recently demonstrated by us [23]. A detailed description of the employed methods can be found in supplementary methods (Supplementary files, supplementary methods).

## Results

### Mononuclear inflammatory response in COVID-19 cardiac tissue

**Fig. 1:**
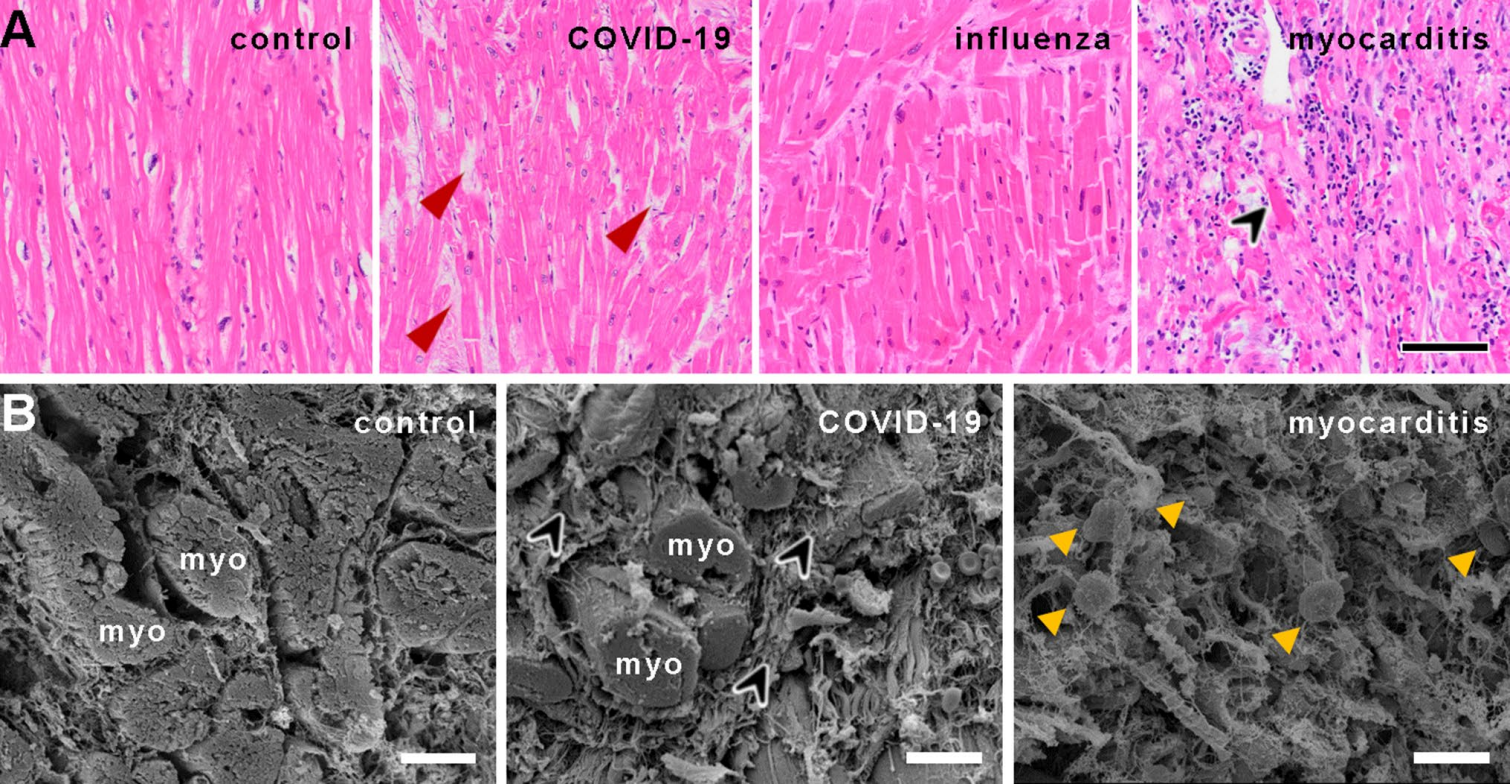
**A** Histological comparison of COVID-19, influenza, and lymphocytic non-influenza myocarditis to non-infected controls. The myocardium of a COVID-19 patient shows no inflammatory infiltrate or cardiomyocyte necrosis with minor interstitial fibrosis (red arrowheads) and minor hypertrophy (COVID-19 patient ID 3). Myocardium of an influenza patient (Influenza patient ID 5) with minor interstitial fibrosis, moderate hypertrophy, and no inflammatory infiltrate or cardiomyocyte necrosis, whereas the myocardium in lymphocytic non-influenza myocarditis (Myocarditis patient ID 5) revealed dense infiltration of the inflammatory cells with single-cell necrosis (black arrowhead). Myocardium of a control patient (Control patient ID 8) displayed no inflammatory infiltrate, necrosis, fibrosis or hypertrophy, H&E staining, Magnification 100x, scale bar 100 µm. **B** Scanning electron micrographs of control heart tissue (left), COVID-19 (center), and lymphocytic myocarditis (right). In COVID-19 heart tissue (center), heart muscle fibers (myo) with slight hypertrophy are surrounded by a meshwork of collagenous fibers (black arrowheads). The orthogonal cardiac muscle orientation seems to be altered compared to the parallel organizations of myocardial strands (myo) in non-infected control heart tissue (left). The myocardial morphology in lymphocytic non-influenza myocarditis (right) is severely disturbed with pronounced edema, sporadic necrosis, and extensive lymphocytic infiltrates (yellow arrowheads), scale bars: 30 µm

Conventional light microscopy of COVID-19 heart samples showed minor inflammatory infiltrates consisting mainly of macrophages in only 1 of 24 cases. The Influenza group showed no significant inflammatory infiltration in all 16 analyzed cases using light microscopy. In contrast, non-influenza/non-SARS-CoV-2 myocarditis cases revealed lymphocytic infiltrates, with minor infiltrates in 4 of the 8 cases, severe lymphocytic infiltrates in 3 of the 8 cases and 1 of the 8 cases with only minimal sparse inflammatory infiltrate with adjacent single-cell necrosis, all but one fulfilling the Dallas criteria for myocarditis (Fig. 1A), while no necrosis was observed in COVID-19, influenza or control tissue. Regarding interstitial fibrosis, no statistically significant (Chi-square test *p* = 0.45) difference was found between the four groups, with COVID-19 (mean score 0.85) displaying the highest and the Influenza group (mean score 0.6) displaying the lowest fibrotic remodeling, respectively (Fig. 1A, B). Cardiomyocyte hypertrophy was most pronounced in the COVID-19 group (mean score 1.2), followed by control cases (mean score 1.1), and with influenza (mean score 0.4) and common viral myocarditis cases (mean score 0.8) showing the least amount of hypertrophy (combined Chi-square test *p* < 0.0001). However, COVID-19 patients were statistically significantly older (73.7 ± 10.8 years) compared to influenza (52.3 ± 15.3 years, *p* < 0.001), common myocarditis (54.8 ± 21.3; p < 0.05), and non-inflamed control patients (54.9 ± 18.0, *p* < 0.05). Immunohistochemistry for SARS-CoV-2 spike and nucleocapsid protein as well as RNA-FISH for SARS-CoV-2 RNA showed no specific signal in the analyzed COVID-19 tissues. However, RT-PCR for SARS-CoV-2 RNA yielded positive results in 17 of the 24 analyzed samples (supplementary Table 1).

**Fig. 2:**
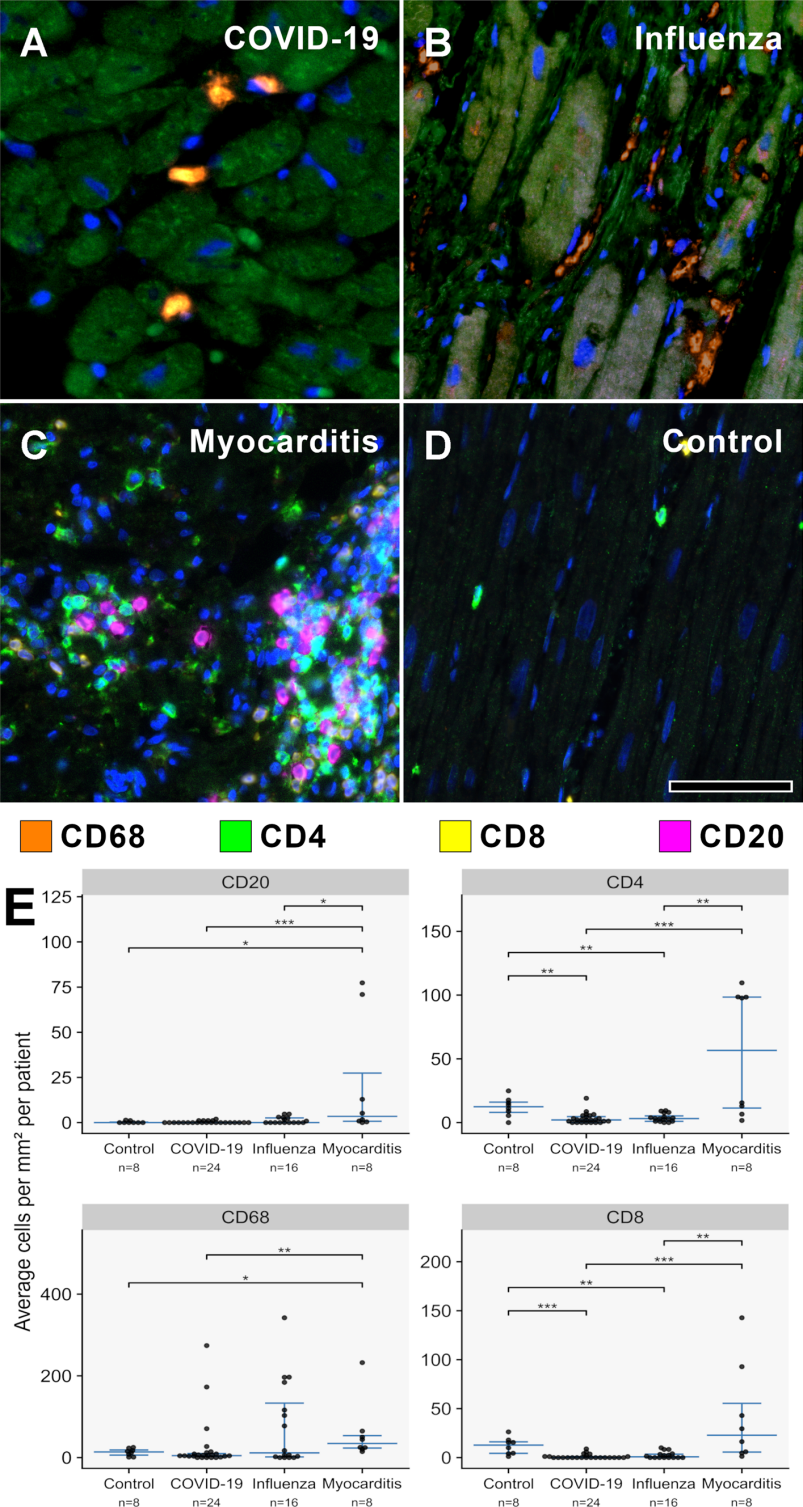
**A**–**D** MPX staining of cardiac tissue depicting CD68 + macrophages in orange, CD4 + T helper cells in green, CD8 + cytotoxic T cells in yellow, and CD20 + B-cells in magenta. All infected hearts (COVID-19, influenza, and lymphocytic non-influenza myocarditis) displayed a prominent infiltrate of CD68 + macrophages. While COVID-19 **A** (COVID-19 patient ID 24) and influenza, **B** (Influenza patient ID 9) hearts showed nearly absent lymphocytic infiltrate, lymphocytic non-influenza myocarditis, **C** (Myocarditis patient ID 5) was characterized by a mixed, T-cell dominated infiltrate. Non-infected control hearts, **D** (Control patient ID 1) showed markedly less inflammatory cells with a mixed population of macrophages and predominant t-cells and only scarce B-cells. Magnification 400x. Scale bars = 100 µm. **E** Histogram of the inflammatory cell infiltrates (CD20, CD4, CD68, CD8). Cell counts are normalized to cells per mm^2^ myocardial tissue. *p < 0.05, **p < 0.01, ***p < 0.001

A more detailed analysis of the inflammatory cell composition showed a marked increase of macrophages (CD68 +) in all infectious groups compared to controls, with influenza cases displaying the most prominent infiltrate (130.2 ± 1.0) with a twofold higher number of macrophages per mm^2^ compared to COVID-19 (65.2 ± 0.8) and common myocarditis (72.5 ± 1.6) (Fig. 2, supplementary table 3). Lymphocytic (CD4, CD8, CD20) infiltrates were scarce in both COVID-19 and influenza cases, whereas common myocarditis cases displayed—besides the prominent macrophage infiltrate—a marked mixed lymphocytic inflammatory infiltrate (Fig. 2, supplementary table 3).

### CD11b + /TIE2 + monocytes/macrophages in COVID-19

**Fig. 3:**
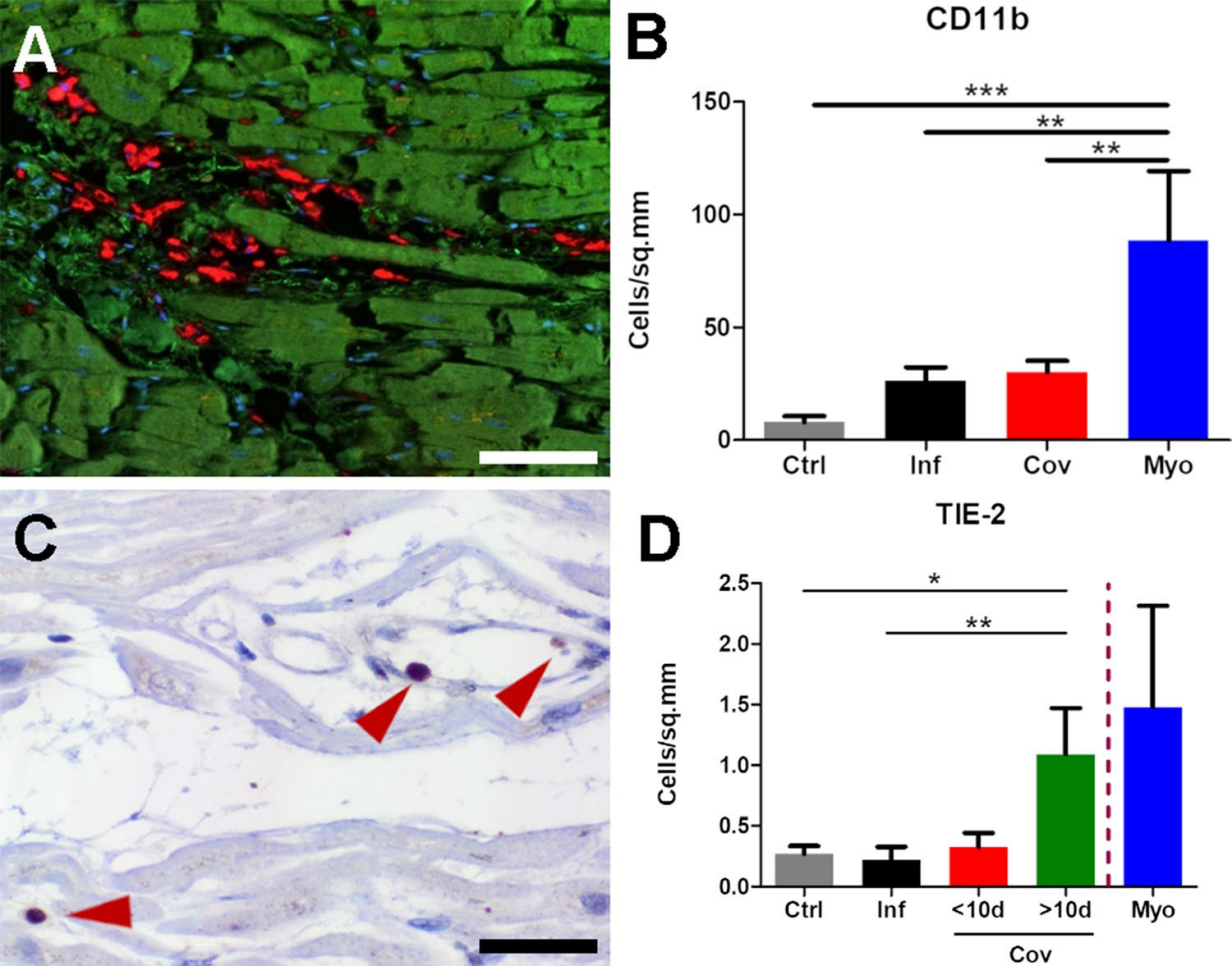
Macrophage expression of CD11b and TIE2. **A** Multiplex immunohistochemistry depicts a diffuse infiltration of CD11b^+^ macrophages (*red*) in the perivascular connective tissue in COVID-19 hearts (COVID-19 patient ID 17), (cardiomyocytes green, nuclei blue), scale bar 100 µm. **B** Bar diagram showing relative infiltration of CD11b^+^ inflammatory cells in non-infected control (Ctrl), influenza (Inf), COVID-19 (CoV), and lymphocytic non-influenza myocarditis (Myo) heart specimens morphometrically assessed by multiplex immunohistochemistry (MPX). Cell counts are normalized to cells per mm^2^ tissue. **C** Immunohistochemical staining against TIE2 demonstrates the perivascular localization of TIE2^+^ cells (red arrowheads) in the myocardium of a COVID-19 patient (COVID-19 patient ID 17), scale bar: 10 µm. **D** Bar diagram showing the infiltration of Tie-2^+^ inflammatory cells in non-infected control (Ctrl), influenza (Inf), COVID-19 (CoV), and lymphocytic non-influenza myocarditis (Myo) heart specimens. Cell counts are normalized to cells per mm^2^ myocardial tissue. Due to the small sample size of lymphocytic non-influenza myocarditis and a high variance among the samples, no statistical tests for significance were carried out. COVID-19 specimens were subdivided into two cohorts of cases with a hospitalization time < 10d and > 10d. *p < 0.05, **p < 0.01, ***p < 0.001

**Fig. 4:**
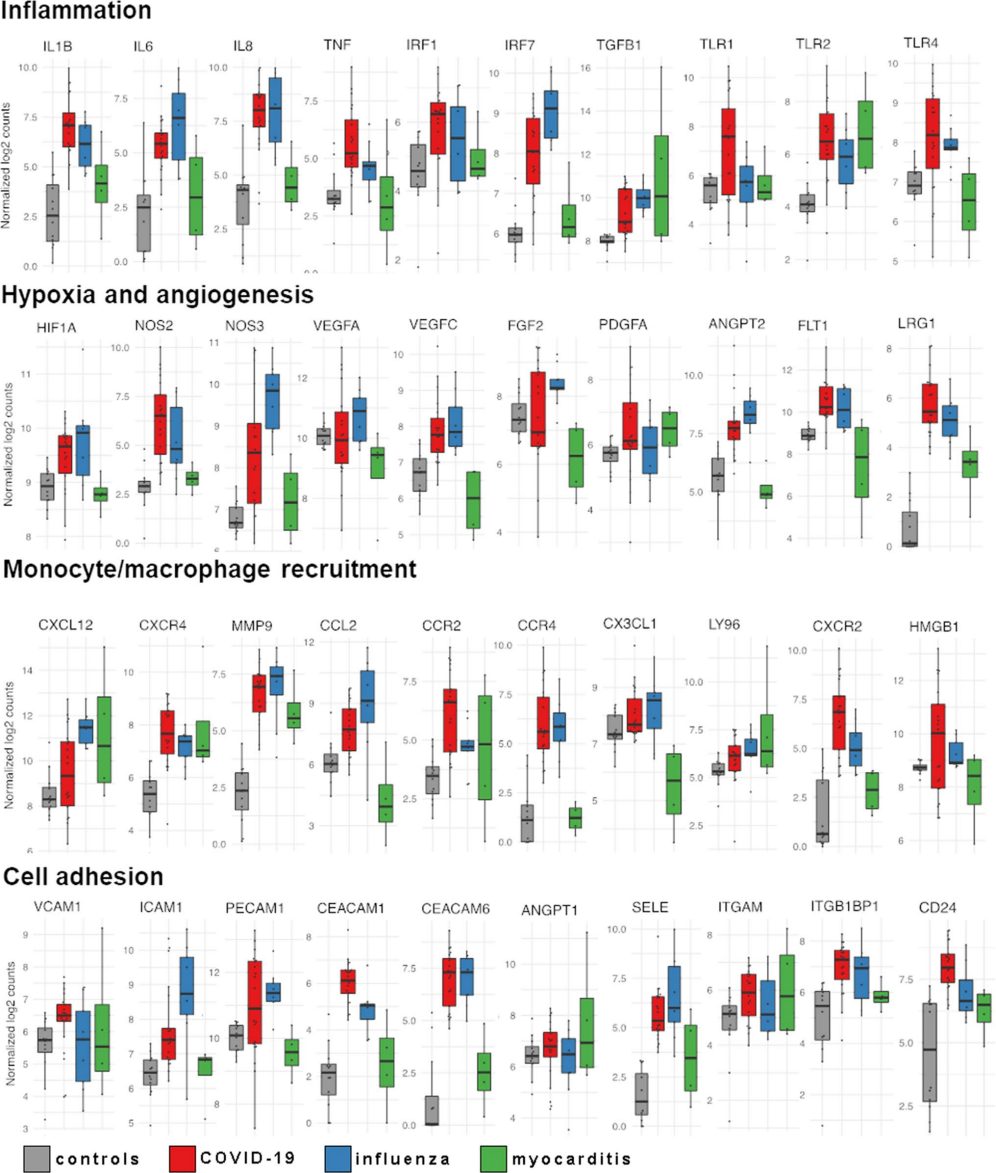
Differential regulation of mRNA expression in the cardiac tissue of COVID-19, influenza, and lymphocytic non-influenza myocarditis cases assessed by multiplex NanoString nCounter analysis system. The boxplots show the expression levels of representing genes of inflammation, hypoxia, angiogenesis, monocyte recruitment, and cell adhesion. Boxplots showing normalized log2 counts of mRNA expression and standard error of the mean, whiskers indicate outliers. Significance was evaluated with Benjamini–Hochberg correction. *FDR < 0.05, **FDR < 0.01, ***FDR < 0.001

Macrophages were the predominant inflammatory cell type in both COVID-19 and influenza cases (Fig. 3A). Regarding macrophage subpopulations, a shift towards an M2 phenotype as indicated by the significant increase in CD16 + CD163 + macrophages in COVID-19 (141.4 ± 129.3 cells per mm^2^) and lymphocytic non-influenza myocarditis (1112.8 ± 1683.4 cells per mm^2^) when compared to influenza (32.6 ± 34.6 cells per mm^2^). Other marker combinations (CD16- CD163 + and CD16 + CD163 + S100A9 + , respectively) did not yield significant differences (Supplemental Table 3). We found a diffuse infiltration of CD11b + -macrophages in the perivascular connective tissue in 18 of 24 COVID-19 heart samples (Fig. 3A, B, supplementary Table 3) to a varying degree. As expected, CD11b expression was found primarily on monocytes and perivascular macrophages (Fig. 3A). As we could find a higher expression of *TIE2* (TEK) in COVID-19 compared to influenza, non-influenza viral myocarditis, and control samples by nanostring analysis, we performed additional immunohistochemical stains demonstrating TIE2 expression primarily on macrophages (supplementary Fig. 1). TIE2-expressing macrophages (TieMs) were present at a significantly higher level in COVID-19 samples (0.7 ± 0.2 cells per mm^2^; Fig. 3C) compared to influenza samples (0.22 ± 0.108 cells per mm^2^; Fig. 3D) and control samples (0.27 ± 0.07 cells per mm^2^, Fig. 3D). Furthermore, immunohistochemical presence of TieMs was elevated more than fivefold in hearts from COVID-19 patients hospitalized later than 10 days after the detection of the infection (1.9 ± 0.6 cells per mm^2^, Fig. 3D) compared to COVID-19 patients hospitalized earlier (0.4 ± 0.1 cells per mm^2^, Fig. 3D). This is supported by an increased expression of Angiopoietin 2 in COVID-19 hearts on a gene expression level (Fig. 4) as well as on protein level, detected by IHC (supplementary Fig. 1), compared to controls.

### Vascular remodeling by intussusceptive angiogenesis contributes to cardiac adverse repair in COVID-19 microangiopathy

**Fig. 5:**
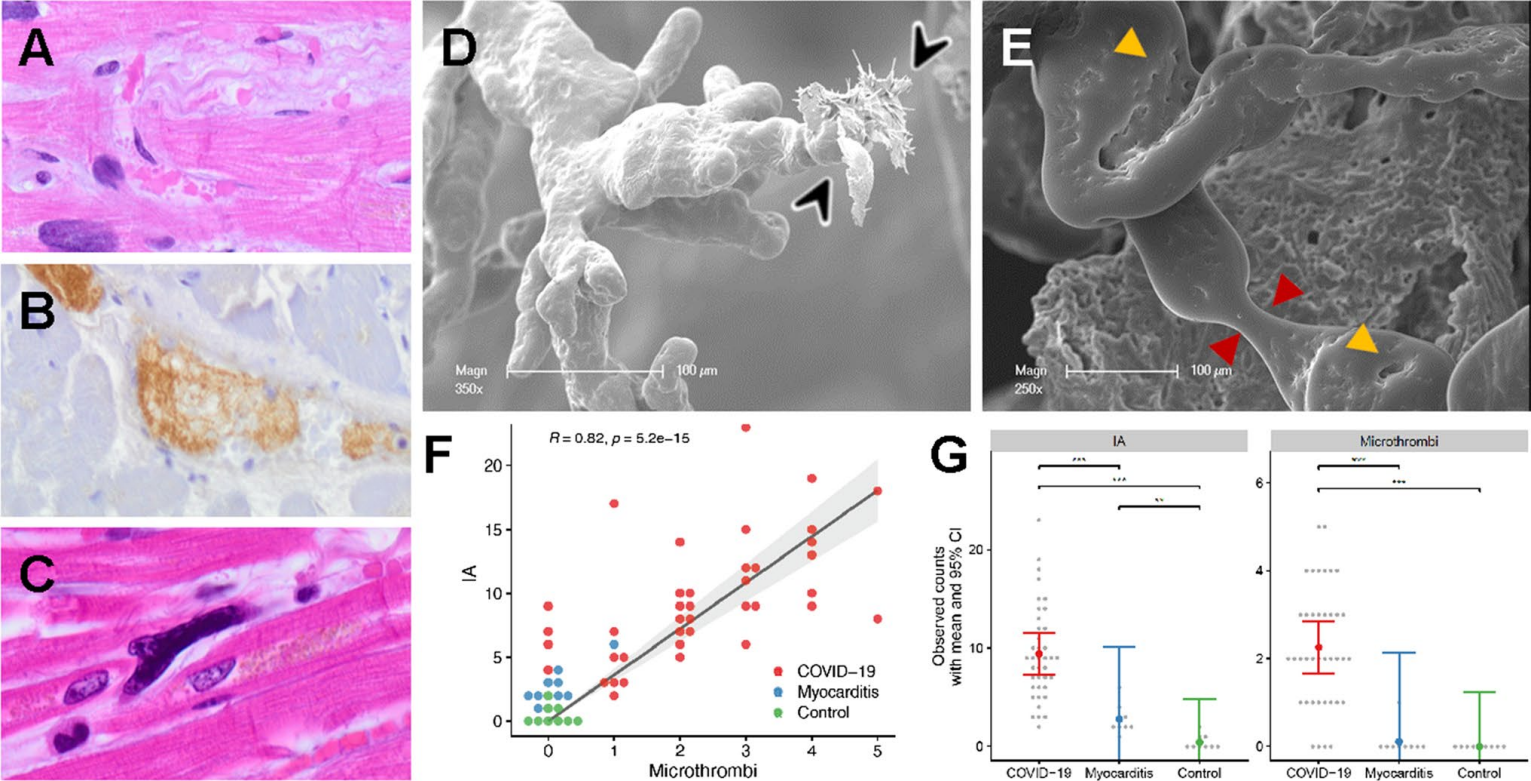
Visualization of ultrastructurally detectable thrombi (uTh) in COVID-19 hearts. **A** H&E staining of a thrombus in a smaller blood vessel in a field of interstitial fibrosis. **B** Immunohistochemical staining against activated fibrin displayed the formation of thrombus in a larger blood vessel. **C** Occasionally, small intracapillary megakaryocytes were observed in COVID-19 autopsy tissue, magnification 400× (COVID-19 patient ID 9). **D**, **E** Scanning electron micrograph of microvascular corrosion casting depicting numerous irregularly dilated and blind-ending vessels with vanishing microvascular hierarchy and micro-extravasation (black arrowheads) indicative for microangiopathy in COVID-19 heart tissue. Cardiac involvement of COVID-19 demonstrates caliber changes with dilated segments and focal vasoconstrictions (red arrowheads). The expansion of vascular plexus by intussusception (yellow arrowheads) is distinctly occurring in the dilated vessel segments, preferably on sites of vessel branching. Scale bars 100 µm. **F** Quantification of visible microthrombi (indicated by premature obliteration of the capillary network (approximate diameter 1–3 µm)) and intussusceptive neoangiogenesis (indicated by the formation of intussusceptive pillars) in COVID-19 and lymphocytic non-influenza myocarditis compared to healthy control tissue. **G** Correlation between the presence of uTh formation and the number of intussusceptive pillar formation in COVID-19 and lymphocytic non-influenza myocarditis compared to non-infected control tissue

**Fig. 6:**
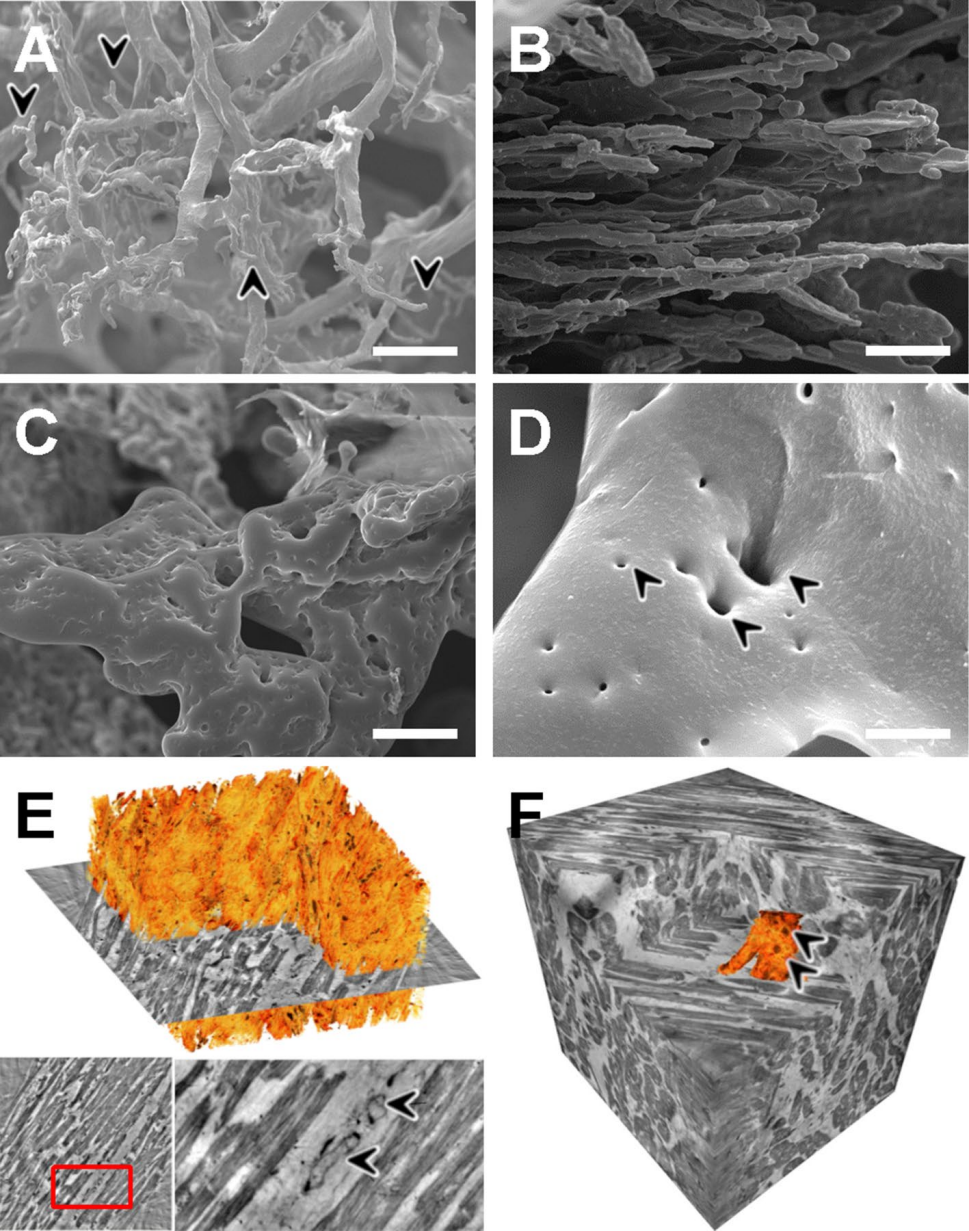
Assessment of the cardiac microvasculature by microvascular corrosion casting and X-ray phase-contrast tomography. Scanning electron micrographs of microvascular corrosion casts revealed in COVID-19 hearts **A** a distorted microvascular architecture with dilated segments and blind-ending stocks (black arrowheads), whereas the vascular architecture of control heart tissue, **B** displayed a regular hierarchical organization (approximate vessel diameter 1–3 µm). **C** Microvascular corrosion casting exposed an irregularly shaped and tortuous microvascular architecture with numerous tiny holes, scale bar 20 µm. **D** Scanning electron micrograph of COVID-19 cardiac vascular plexus highlights the confluent expansion of transluminal intussusceptive pillars (black arrowheads) at a branching point, often seen as doublets and triplets, scale bar 5 µm. **E** Volume rendering of a tomographic reconstruction obtained by synchrotron-radiation-based X-ray phase-contrast tomography highlighting the mild interstitial fibrosis (orange) in COVID-19 heart tissue. In the magnification of the presented 2D-slice (marked by a red rectangle) a nucleus of an endothelial cell and an intraluminal pillar (black arrowheads) are visible. **F** Volume segmentation of microvasculature (depicted in orange) in phase-contrast synchrotron-radiation-based-X-ray tomographs demonstrate the altered microvascular architecture (Arrowheads) in COVID-19 hearts compared to the parallel alignment of coronary plexus in control hearts. The reconstructed dataset shown in **F** has been recorded at 167 mm voxel size. A cube of 350 µm side length is shown

Using conventional light microscopy and immunohistochemical staining for fibrin, only scarce small vessel thrombi and no large vessel thrombi could be demonstrated in COVID-19 hearts (Fig. 1A), while two heart samples in the influenza group showed large vessel thrombi without thrombi in small vessels (Fig. 5A–C). Interestingly, contrary to the light microscopy findings, SEM imaging of corrosion casts of COVID-19 hearts showed a marked increase in the presence of ultrastructurally detectable thrombi (uTH), as indicated by abrupt breakoff of capillaries (Fig. 5D and Fig. 6A). These capillaries had a diameter of 1–3 µm, too small to be reliably detectable in conventional light microscopy, especially in autopsy material. Moreover, COVID-19 hearts showed an altered vascular architecture with a loss of vascular hierarchy, tortuous arrangement (Fig. 6A), and irregular sinusoidal vessel networks with frequent vessel diameter changes compared to the paralleled alignment of cardiac microvascularity in the healthy controls (compare Fig. 6B, D). Furthermore, the presence of transluminal intussusceptive pillars indicated by small holes in vascular corrosion casts (Fig. 5E and Fig. 6D), partly as doublets and triplets, at numerous vessel branches (Fig. 6D), could be found in COVID-19 hearts, but not in non-influenza myocarditis cases. Quantification of corrosion casts proved that non-influenza viral myocarditis cases had significantly fewer intussusceptive pillars (2.8 ± 3.7 SE) and uTh (0.1 ± 1.0 SE) compared to COVID-19 hearts (9.4 ± 1.0 SE and 2.3 ± 0.3 SE, respectively, Fig. 5F). Of note, we found a positive correlation between the presence of microthrombi and the occurrence of intussusceptive pillars in COVID-19 hearts (Fig. 5G). Corrosion casts of control hearts did not show signs of uTH or intussusceptive pillars (Fig. 6B). Archived material of the 2009 influenza pandemic suitable for corrosion casting was not available.

Phase-contrast synchrotron radiation tomographic microscopy (SRXTM) identified the spatial coincidence of morphological aspects of disturbed, feathered course of heart fibers, mild interstitial fibrosis, and the occurrence of intussusceptive pillars (Fig. 6E). Moreover, volume segmentation of SRXTM tomographs demonstrated vascular alterations in COVID-19 hearts at the level of the afferent, large-caliber vessels (Fig. 6F), compared to regular vascular architecture in the analyzed control tissue.

### Differential regulation of myocardial mRNA expression and associated biological functions in COVID-19, influenza, and myocarditis

We performed a digital multiplexed gene expression analysis using NanoString nCounter® technology. In COVID-19 hearts, we identified significant upregulation of pro-inflammatory genes *IL1B*, *IL-6*, *IL8*, and the toll-like receptor *TLR2* (Fig. 4). Moreover, significant upregulation of hypoxia and angiogenesis-related genes as documented by *VEGFC*, *FLT1,* and *NOS3* was found (Fig.4, FDR < 0.05). Gene expression analysis showed a significant upregulation of genes associated with monocyte recruitment such as *CXCR4*, *MMP9*, *CCR2*, *CXCR2*, and *MYD88* (Fig. 4, FDR < 0.001). Moreover, cell adhesion markers such as *ICAM1*, *PECAM1*, and *SELE* were markedly, and significantly, upregulated in COVID-19, influenza, and non-influenza viral myocarditis (Fig. 4, FDR < 0.001). TIE2 was not ubiquitously upregulated in COVID-19, influenza, and non-influenza viral myocarditis compared to controls, but 10 of the 24 COVID-19 hearts displayed a marked upregulation of up to tenfold (supplementary material).

**Fig. 7:**
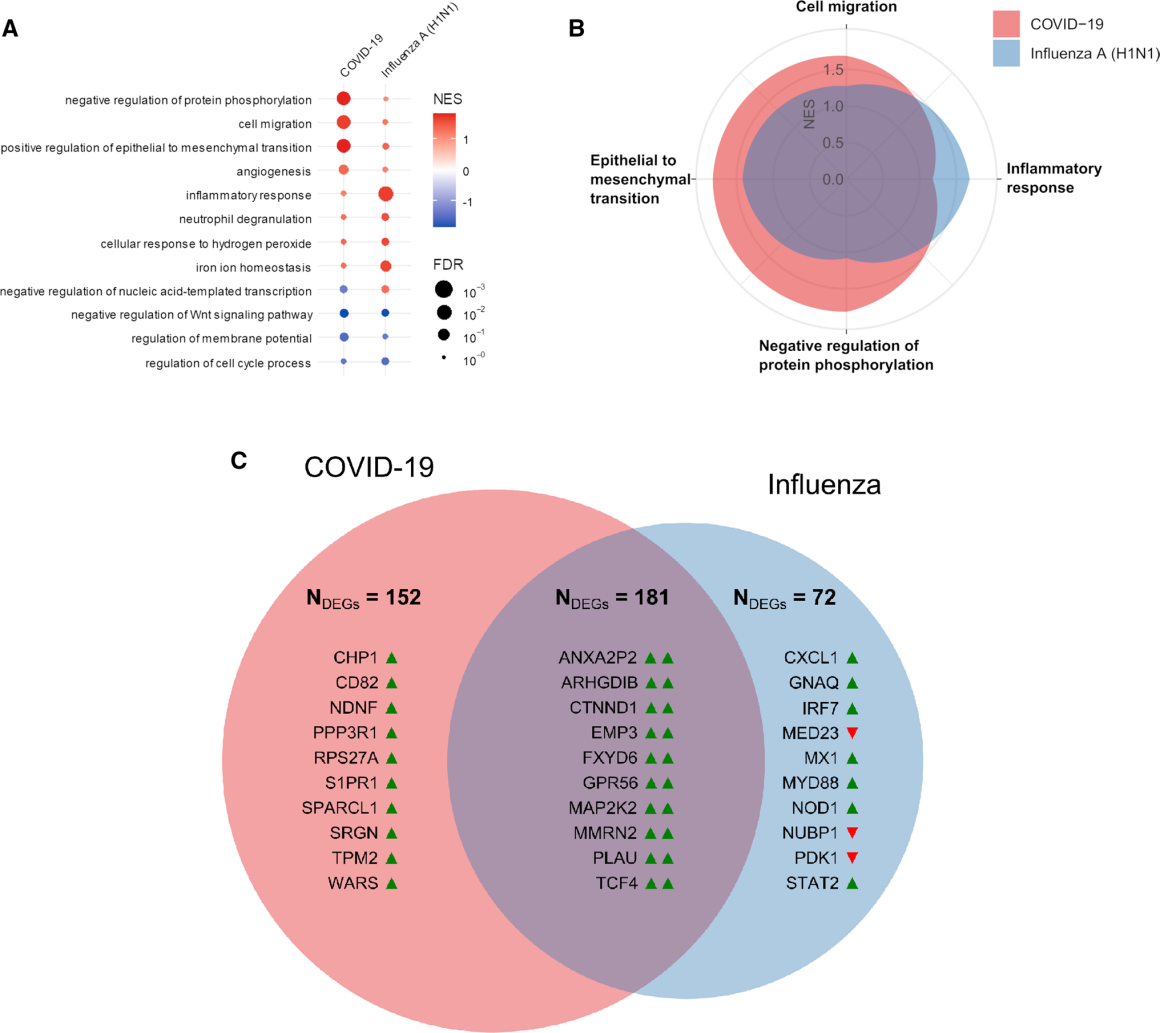
Functional gene expression analysis of COVID-19, influenza, and lymphocytic non-influenza myocarditis heart tissues. **A** Functional Pathway analysis via gene set enrichment analysis against the Gene Ontology aspect biological functions highlights the differential functional gene expression in the heart tissue of COVID-19 and influenza patients. The activation of biological functions in cardiac injury patterns compared to healthy controls was predicted for each sample. Color indicates up- (red) and down (blue)-regulation; circle size depicts FDR. Only significantly up-or down-regulated pathways are shown. **B** Spider-Plot depicting the enrichment of biological functions from Gene Ontology based on gene expression data of COVID-19 and influenza heart samples as compared with expression in non-infected control specimen. The y-axis shows the normalized enrichment scores (NES) on a scale from 0 to 2. **C** Venn diagram of statistically differentially expressed genes of COVID-19 and influenza heart samples as compared with expression in controls in both disease groups (Student’s t test, controlled for the familywise error rate with a Benjamini–Hochberg false discovery rate threshold of 0.05). Up-regulation and down-regulation of genes are indicated by colored arrowheads suffixed to the gene symbols (green denotes upregulation, red denotes down-regulation). Numbers given are the total of differentially regulated genes, displayed are the top 10 up- or down-regulated genes. Note that there were no differentially expressed genes for lymphocytic non-influenza myocarditis

In addition to canonical pathways, differentially expressed genes were categorized to related functional pathways via gene set enrichment analysis against the Gene Ontology (GO) aspect biological functions. A complete list of GO annotations and a complete list of the analyzed genes is given in the Supplementary material. Gene expression data analysis of 797 analyzed genes showed clear differences between COVID-19 and influenza cases, but not between COVID-19 cases and non-influenza lymphocytic myocarditis (Fig. 7A). While influenza cases were primarily characterized by pro-inflammatory signaling and classical anti-viral response gene expression (e.g., *BCL6*, *DLL4*, *GDF15*, *PTX3,* *HMGB2,* and *IL-8*). COVID-19-associated gene expression showed significant upregulation of angiogenesis, cell migration, and epithelial-mesenchymal-transition (EMT) pathways in the ingenuity pathway analysis (Fig. 7B).

Comparative gene analysis demonstrated twice as much differentially expressed genes in COVID-19 compared to influenza (152 genes in COVID-19 vs 72 genes in Influenza) (Fig. 7C).

## Discussion

**Fig. 8:**
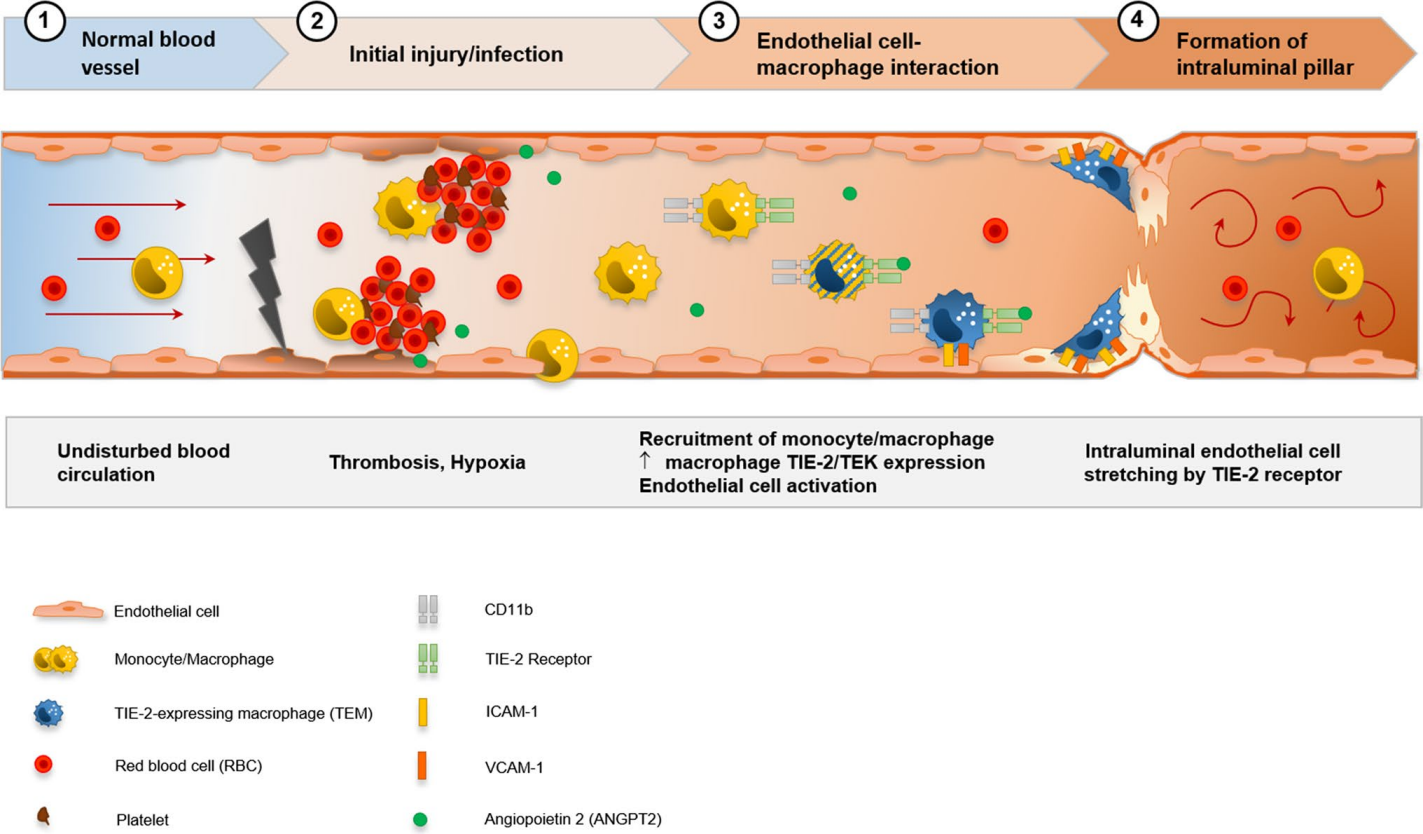
Visualization of the hypothesis of CD11b + /TIE2 + monocytes/macrophages recruitment and incorporation, and intussusceptive angiogenesis in the cardiac vasculature in COVID-19. SARS-CoV-2-related endothelial dysfunction results in thrombotic microangiopathy in cardiac capillaries and tissue hypoxia. Endothelial cells induce the recruitment of monocytes/macrophages to the site of injury by upregulation of adhesion-molecules and activation of SDF-1/CXCR4 signaling. TIE2 + monocytes/macrophages are activated by increased levels of angiopoietin 1 and adhere locally in response to angiopoietin 2 to endothelial cells. Formation of an intussusceptive pillar is achieved by intraluminal stretching of endothelial cells under the help of adherent TIE2 + monocytes/macrophages resulting in the division of a single capillary altering the cardiac microvasculature

We previously reported a prominent vasculocentric inflammation and its sequelae in COVID-19 lung injury [4], which result from direct injury of the pulmonary endothelium. Here, for the very first time, we comprehensively assessed and compared the molecular, morphological, and ultrastructural aspects of injury patterns in cardiac tissue of patients, who succumbed to COVID-19, influenza and non-influenza lymphocytic myocarditis. We propose that a similar vascular tropism [24] with subsequent endothelial damage resulting in marked alterations of the cardiac microvasculature, including an increased number of uTh and local hypoxia, characterize the cardiac involvement in COVID-19 (Fig. 8).

COVID-19-associated cardiac injury is discussed as both direct and indirect injury to the myocardium. Although cardiomyocytes express significant levels of ACE2, which serves as SARS-CoV-2 receptor [6], the lack of TMPRSS2, a cell-membrane-based protease needed for conformation changes in the spike protein of SARS-CoV-2 and cell membrane fusion [7], likely hampers direct cardiomyocyte infection. Another hypothesis for the cardiac involvement in COVID-19 implicates the systemic release of pro-inflammatory cytokines (e.g., IL-1, IL-6, TNF-α, IFN-γ, and MIP) and the subsequent “cytokine storm” as the main culprit with subsequent increased vascular wall permeability and myocardial edema [11]. Consistently, we found signs of endothelial damage and endothelial activation in the form of increased gene expression pathways regarding EMT/EndoMT in COVID-19 hearts accompanied by an upregulation of tissue hypoxia-related pathways and the formation of uTh. Here, it should be noted that in our study, EMT might rather reflect EndoMT, two largely overlapping pathways with the latter not reflected in Ingenuity`s pathway analysis [25, 26].

Vascular injury and tissue hypoxia lead to an activation of local macrophages [27] and drive transcriptional profiles associated with the recruitment of circulating monocytes in COVID-19, as documented by the observed marked increase in tissue macrophages and the upregulation of *CCR2*, *CYCR2*, *CXCR4,* and *MYD88.* The recruitment of bone marrow-derived mononuclear cells has been identified in a variety of cardiovascular diseases, including atrial fibrillation [28], myocardial infarction [29], and heart failure [28], but also in cardiac fibrosis [30] and extra cardiac diseases, e.g., bronchiolitis obliterans in the lung [31]. Monocyte recruitment is also supported by tissue-resident CCR2 + macrophages—especially CXCR4 + /CD11b + monocytes via MYD88-dependent and SDF-1/CXCR4 pathway-associated mechanisms, resulting in a release of inflammatory cytokines. Additionally, monocytes are hypothesized to be involved in angiogenesis either via the depletion of proangiogenic growth factors (e.g., *VEGF*, *FGF2,* or *eNOS*) or by mimicking endothelial progenitor cells (EPCs) and becoming incorporated as endothelial cells [32]. Our transcriptional data provide evidence that significantly higher levels of hypoxia-related and proangiogenic genes (*HIF1a*, *VEGFA*, *FGF2*, *eNOS*, *SOX17,* and *LRG1*) promote neovascularization at the site of increased cardiac ischemia.

Thus, macrophages are the main responders involved throughout the pathomechanistic sequence [33], from recruitment of circulating monocytes over the mediation of cardiac inflammation, to irreversible tissue remodeling of the fibrovascular interface in the form of intussusceptive angiogenesis.

Pronounced neovascularization in form of CD11b/TIE2 +—macrophage associated intussusceptive angiogenesis, as previously described in other organs [34], has been our most intriguing observation in cardiac remodeling triggered by SARS-CoV-2 infection.

Intussusceptive (non-sprouting) angiogenesis is a highly dynamic process observed within minutes to hours after a stimulus without requiring cell proliferation [35] and is described also in the early phases of the disease in COVID-19 lungs [4]. In this process, the formation of a transluminal pillar is crucial for the duplication and expansion of the vascular plexus. Intussusceptive angiogenesis and similar hemodynamic alterations have previously been observed in atherosclerosis [36], inflammatory diseases [37], malignancies [38], and in parvovirus B19 viral myocarditis [39]. Additionally, intussusceptive angiogenesis plays a pivotal role in fibrotic remodeling, for example in interstitial lung diseases [34] as well as in protection against prothrombotic endothelial dysfunction in COVID-19 [40].

The molecular and mechanical factors involved in the rapid expansion of intussusceptive angiogenesis are, thus, far poorly understood, in part, because in vitro models for intussusception are missing. However, it is known that SDF-1/CXCR4 signaling is involved in the blood vessel formation and remodeling by intussusception [41]. In a parabiosis model, we previously reported that blood-borne migrating CD11b + monocytes significantly contribute to lung regeneration and angiogenesis via intussusception [42]. Mechanistically, this occurs via SDF-1 mediating the arrest of CD34 + endothelial progenitor cells on the vascular endothelium under shear flow by the action of the integrins VCAM and ICAM [43]. TIE2-expressing cells then promote the incorporation, maturation, and vascular stabilization of the bone marrow-derived monocytes [44]. Growing evidence implicates a pivotal role for the SDF-1/CXCR4 axis in myocardial repair, especially in myoangiogenesis, characterized by recruitment and engraftment of bone marrow-derived mononuclear cells after acute myocardial infarction [45], in cardiomyopathy [46], and negatively predicting mortality in viral myocarditis [47]. Our results support these findings by identifying a significant infiltrate of CD11b + /TIE2 + macrophages with concurrent increased intussusceptive angiogenesis and a significant upregulation of *CXCR4*, *SDF-1,* and *MMP9*.

It should be noted that our study has some limitations. In the literature, Tie-2 is described as a marker of endothelial cells. However, the antibody clone we used worked on a subset of macrophages but not consistently on endothelial cells of the autopsy material likely due to autolysis artifacts. Due to technical shortcomings, detailed cardiovascular parameters were not available for all patients, and not all analyses could be performed on each case. For technical reasons, ultrastructural analysis is feasible only in larger tissue samples from autopsies, thus, restricting our study to patients with intensive care treatment and fatal outcomes. Additionally, the COVID-19 cohort was significantly older, which potentially influenced parameters such as cardiac fibrosis or hypertrophy. Lastly, due to a limited cohort size as well as incompletely reported clinical findings, consistent conclusions on individual attributability cardiac symptoms to underlying molecular alterations were unfeasible. Despite these restrictions, our results define a distinct and novel cardiac manifestation of severe COVID-19.

To summarize, our findings suggest that (I) cardiac involvement in COVID-19 does not present as conventional viral myocarditis defined by mononuclear infiltrates and myocyte damage, but rather as a perivascular infiltration by a special subpopulation of CD11b + /TIE2 + macrophages contributing to cardiac neoangiogenesis and remodeling of the fibrovascular interface. (II) COVID-19 cardiac involvement is substantially underappreciated in conventional light microscopy and its adequate diagnosis requires a multimodal approach. (III) Our comparative morphological and transcriptional data indicate that the irreversible remodeling due to intussusceptive angiogenesis—based on our experience in other organs—is the main driver for a specific COVID-19-induced cardiac injury. It is facilitated by interactions between the incorporated of blood-borne CD11b + /TIE2 + monocytes/macrophages and damaged and activated endothelial cells in COVID-19 hearts (Fig. 8). A systematic follow-up of non-fatal COVID-19 and long-COVID cases appear highly warranted for the detection of possible post-acute cardiac symptoms and fibrotic remodeling also in mild cases of disease due to the irreversible alterations of the cardiac vasculature.

## Supplementary Information

Below is the link to the electronic supplementary material.

Supplementary file1 (XLSX 366 kb)

Supplementary file2 (XLSX 498 kb)

Supplementary file3 (TIF 2827 kb)

Supplementary file4 (DOCX 59 kb)

Supplementary file5 (TIF 14928 kb)

## Data Availability

Additional Data will be made available on reasonable request**.**

## Abbreviations

ACE2: Angiotensin-converting enzyme 2
BCL6: B-cell lymphoma 6
BMDCs: Bone marrow dendritic cells
BM-MNCs: Bone marrow-derived mononuclear cells
B-βCoV: B beta-coronavirus
CCR2: C–C chemokine receptor type 2
CD4: Cluster of differentiation molecule 4
CD8: Cluster of differentiation molecule 8
CD11b: Cluster of differentiation molecule 11b
CD16: Cluster of differentiation molecule 16
CD20: Cluster of differentiation molecule 20
CD34: Cluster of differentiation molecule 34
CD68: Cluster of differentiation molecule 68
CD163: Cluster of differentiation molecule 163
CK-MB: Creatine kinase myocardial band
COVID-19: Coronavirus disease 2019
CXCL12: C-X-C motif chemokine 12
CXCR2: C-X-C chemokine receptor type 2
CXCR4: C-X-C chemokine receptor type 4
DLL4: Delta-like canonical notch ligand 4
E gene: Encoding envelope protein
EMT: Epithelial-mesenchymal transition
EndoMT: Endothelial-mesenchymal transition
EPCs: Endothelial progenitor cells
EvG: Elastica van giesson
FDR: False discovery rate
FFPE: Formalin-fixed paraffin-embedded
FGF2: Fibroblast growth factor 2
FISH: Fluorescence in situ hybridization
FLT1: Fms-related receptor tyrosine kinase 1
GDF15: Growth/differentiation factor 15
H1N1: Hemagglutinin-1-neuraminidase-1
HE: Hematoxylin and Eosin
HIF1α: Hypoxia-inducible factor 1-alpha
HMGB2: High-mobility group box 2
ICAM1: Intercellular adhesion molecule 1
ICU: Intensive care unit
IFN-γ: Interferon-gamma
IgG: Immunoglobulin G
IHC: Immunohistochemistry
IL-1: Interleukin-1
IL1β: Interleukin 1 beta
IL-6: Interleukin 6
IL8: Interleukin 8
LRG1: Leucine-rich alpha-2-glycoprotein 1
LVEF: Left ventricular ejection fraction
MIP: Macrophage inflammatory proteins
MMP9: Matrix metallopeptidase 9
MPX: Multiplexed immunohistochemistry
MRI: Magnetic resonance imaging
mRNA: Messenger ribonucleic acid
MYD88: Myeloid differentiation primary response 88
NOS3: Nitric oxide synthase 3
NT-proBNP: N-terminal prohormone of brain natriuretic peptide
P: Probability
PAS: Periodic acid schiff
PCR: Polymerase chain reaction
PECAM1: Platelet and endothelial cell adhesion molecule 1
PTX3: Pentraxin 3
RNA: Ribonucleic acid
ROIs: Regions of interest
RT-PCR: Reverse transcription polymerase chain reaction
RVEF: Right ventricular ejection fraction
S100A9: S100 calcium-binding protein A9
SARS-CoV-2: Severe acute respiratory syndrome coronavirus type 2
SD: Standard deviation
SDF-1: Stromal cell-derived factor 1
SE: Standard error
SELE: Selectin E
SEM: Scanning electron microscope
Sox17: SRY-box transcription factor 17
SRXTM: Synchrotron radiation tomographic microscopy
TIE2: Angiopoietin-1 receptor
TieMs: TIE2-expressing macrophages
TLR2: Toll-like receptor 2
TMPRSS2: Transmembrane protease serine subtype 2
TNF-a: Tumor necrosis factor alpha
uTH: Ultrastructurally detectable thrombi
VCAM: Vascular cell adhesion molecule
VEGFA: Vascular endothelial growth factor A
VEGFC: Vascular endothelial growth factor C
