# Supplementary material for: Inflammation and vascular remodeling in COVID-19 hearts
Source: Angiogenesis. 2022 Nov 12;26(2):233–48. doi: 10.1007/s10456-022-09860-7 (PMC9660162; doi:10.1007/s10456-022-09860-7)
Supplement: Supplementary file 4 — Supplementary file4 (DOCX 59 kb) [file 10456_2022_9860_MOESM4_ESM.docx]

**Supplementary files**

**Supplementary methods**

**Histopathologic Analysis**

Heart FFPE samples of COVID-19, influenza A, and non-influenza myocarditis were examined via Hematoxylin&Eosin (HE), Elastica van Giesson (EvG), and Periodic Acid Schiff (PAS) staining. For qualitative and quantitative assessment of vascular thrombi by conventional histology HE, PAS, and EvG stains were visually assessed at high magnification by an experienced pathologist. Areas of comparable morphology for each heart sample were analyzed from low (20x) to high magnification (400x) using immunohistochemical stains of activated fibrin as additional adjuncts. Visible inflammatory infiltrate as well as large vessel and small vessel thrombi were assessed as present or absent, interstitial fibrosis and hypertrophy were semiquantitatively graded from mild over moderate to severe.

**Standard Immunohistochemistry**

Immunohistochemistry was performed on formalin-fixed paraffin-embedded (FFPE) tissue. Briefly, FFPE samples were serially sectioned (2µm thickness). After de-paraffinization using xylene for 10 min. twice, followed by rehydration using the decreasing concentration of ethanol, the sections were subjected to heat-induced epitope retrieval in respective antibody buffer. The sections were stained with the following antibodies using the manufacturer’s protocol of ZytoChem Plus HRP Polymer Kit (Zytomed systems) with DAB (3, 3’-Diaminobinzidine) solution. For double- immunohistochemistry stainings, sections were stained with a primary antibody-cocktail of anti CD31 and anti-TIE-2 using the manufacturer’s protocol of ZytoChem-Plus AP Polymer-Kit (Zytomed systems) with AP (alkaline phosphatase) solution and ZytoChem Plus HRP Polymer Kit (Zytomed systems) with DAB (3, 3’-Diaminobinzidine) solution. After the staining Eukitt® mounting media was used as an adhesive and a sealant.

| **S.No.** | **Antibody** | **Company** | **Cat. No.** | **Dilution** |
| --- | --- | --- | --- | --- |
| **Primary Antibodies** | | | | |
| 1 | Anti-TIE2 | Sigma-Aldrich | SAB4502942 | 1:500 |
| 2 | Anti-CD11b | Abcam | ab 133357 | 1:2000 |
| 3 | Anti-CD68 | Dako | M0876 | 1:750 |
| 4 | Anti-CD4 | Zytomed | 503-3350 | 1:50 |
| 5 | Anti-CD8 | Dako | M7103 | 1:600 |
| 6 | Anti-CD20 | Dako | M0755 | 1:1000 |
| 7 | Anti-CD163 | Cellmarque | 163M-17 | 1:50 |
| 8 | Anti-CD16 | Santa Cruz | sc-20052 | 1:2000 |
| 9 | Anti-S100A9 | abcam | ab63818 | 1:16000 |
| 10 | Anti-CD163 | Cellmarque | 163M-17 | 1:50 |
| 11 | Anti-CD31 | Roche | 760-4378 | RTU (12µg/mL) |
| **Secondary Antibodies** | | | | |
| 7 | Opal520 | Akoya Biosciences | FP1487001KT | 1:100 |
| 8 | Opal540 | Akoya Biosciences | FP1494001KT | 1:100 |
| 9 | Opal570 | Akoya Biosciences | FP1488001KT | 1:100 |
| 10 | Opal650 | Akoya Biosciences | FP1496001KT | 1:100 |

**Multiplex Immunohistochemistry**

FFPE sections from each group (SARS-CoV-2 infected patients n=24, influenza A infected patients n=16, common viral myocarditis n=8, control n=9) were stained with the manual Opal 7-Color IHC Kit (Akoya Biosciences, Marlborough, MA) as described by the manufacturer[1].

The primary antibodies CD4, CD8 , CD68,CD20 CD16, CD163 and S100A9 were combined in sequence with the opal fluorophores Opal520, Opal570, Opal540, Opal570, Opal620 and Opal650. The nuclei were counterstained using Spectral DAPI. The sections were scanned with the Mantra 2™ System (Akoya Biosciences, Marlborough, MA) or VECTRA automated quantitative pathology imaging system (Akoya Biosciences, Marlborough, USA). At least 10 Regions of Interest were selected as representative for the entire tissue section. Cell counting was either performed manually in samples with significant autofluorescence or automatically detected using the InForm software. From each cohort, multispectral images of one patient were used for training and manually annotated using the above-mentioned phenotype. This algorithm was then deployed on all samples to detect the above-mentioned phenotypes. Measurement outputs of the inForm-Software were analyzed using the phenoptr and phenoptrReports packages in R version 4.0.3.

**SARS-CoV-2 Detection**

Immunohistochemical detection of SARS-Cov-2 proteins was performed on tissue sections from FFPE embedded tissue using IgG fractions of a rabbit anti-SARS-CoV-2 spike protein antiserum (40150-R007 Sino Biological, Peking, China) at a dilution of 1:50 and monoclonal mouse anti-nucleocapsid antibody (40143-MM05, Sino Biological, Peking, China) at a 1:100 dilution and detected using DAB detection kit (760-500, Ventana Medical Systems, Inc., Tucson, USA). Fluorescence in situ hybridization (FISH) was performed using the same samples as described for Immunohistochemistry using the HuluFISH probe against SARS-CoV-2 (R-0101-020-OR, MetaSystems, Altlusheim, Germany) following the instructions of the manufacturer.

RNA was extracted from FFPE tissue using a Maxwell® 16 LEV RNA FFPE Purification Kit (Promega GmbH, Walldorf, Germany) on the Maxwell® 16 IVD instrument (Promega GmbH) or with the ReliaPrep™ FFPE Total RNA Miniprep System (Promega GmbH) according to the manufacturer's instructions. We stored the RNA samples at -80°C until further processing. TaqMan™ Fast 1-Step Master Mix (Thermo Fisher Scientific GmbH, Dreieich, Germany) was used for the qualitative detection of the *E* gene (encoding envelope protein) of lineage B beta-coronavirus (B-βCoV) by a primer (0.4 μM) and probe (0.2 μM) set labeled with fluorescent reporters and quencher dyes. TaqMan® Exogenous Internal Positive Control reagents (Thermo Fisher Scientific GmbH, Dreieich, Germany) as internal PCR controls. RT-PCR was performed as previously described[2, 3]. Briefly, RNA was reversely transcribed (50°C for 10 min) and amplified with the reaction mixture at 95°C for 20 s and followed by 45 cycles of 95°C for 3s and 58°C for 30s. Amplirun® SARS-CoV-2 RNA control (Bestbion dx GmbH, Cologne, Germany) provided with 13000 viral RNA copies μL-1 was used to determine the number of viral RNA copies µL-1. Virus positive cases were defined as yielding either positive immunohistochemistry, RNA-FISH signals and/or ct-values <40.

**Microvascular Corrosion Casting**

From the available formalin-fixed unembedded tissue, 13 COVID-19 samples, 3 lymphocytic myocarditis samples and 3 control samples were suitable for vascular corrosion casting. Samples were derived from left ventricular myocardium. The supplying vessels were cannulated through the right ventricle with an olive-tipped cannula and perfused with 10mL saline at 37°C followed by 10mL of a buffered 2.5% glutaraldehyde solution (Sigma) at pH 7.4. After casting the microcirculation with 10mL of polyurethane-based casting resin PU4ii (vasQ-tec, Zurich, Switzerland) and caustic digestion, the microvascular corrosion casts were imaged after coating with gold in an argon atmosphere with a Philips ESEM XL30 scanning electron microscope as described[4]. For quantitative analyses, scanning electron micrographs of microvascular corrosion casts were used for morphometry of parameters defining the alterations of the microvascular unit, namely the oocurence of intussusceptive angiogenesis and microthrombi. For this, all features of intussusceptive angiogenesis were identified in the vascular cast as tiny holes and small capillary loops whose larger diameter between 2 and 5 μm. The quantitation of features was expressed as numerical density per vessel area [13]. Microthrombi were identified as discontinuation of the vessel course or obstructions in the vascular unit and expressed as numerical density per vessel area (**Supplemental Figure 2**).

**RNA Extraction and Gene Expression Analysis**

RNA from FFPE-material of all included cases was isolated using the Maxwell® RNA extraction system (Promega, Madison, Wisconsin) and following quality control via Qubit analysis (ThermoFisher, Waltham, Massachusetts) used for further analysis. All mRNA expression data were obtained via the nCounter® Analysis System (NanoString Technologies, Seattle, WA) using the PanCancer Progression Panel and Inflammation Panel including 967 genes of which 797 genes were analyzed; 170 dropped out due to low signal. Normalization of raw counts was performed using the nSolver™ analysis software version 3.0 (NanoString Technologies, Seattle, WA) and a modified version of the nCounter® advanced analysis module (version 1.1.5). The normalization process included positive normalization (geometric mean normalization factor against positive control lanes), negative normalization (arithmetic mean background subtraction against negative control lanes) and reference normalization (geometric mean normalization factor) using the nSolver Analysis Software version 4.0. Statistical testing (Student’s *t-*tests) and a subsequently calculated false discovery rate (FDR) threshold of 0.05 were used to identify significant changes in gene expression[5].

**Functional Analysis**

To gain insight into the regulation of physiological functions conveyed by the mRNA expression analysis, we made use of a gene set enrichment analysis against the Gene Ontology database[6–8]. We considered false discovery rates significant according to the following levels of confidence: FDR<0.05 (*), FDR <0.01 (**) and FDR <0.001 (***).

**Synchrotron radiation tomographic microscopy (SRXTM)**

FFPE paraffin blocks from eleven COVID-19 and five control heart samples were investigated by phase-contrast X-ray tomography at the GINIX endstation of the P10 beamline at the PETRAIII storage ring 104 (DESY, Hamburg), using an undulator synchrotron beam of 13.8keV, focused by KB-mirrors and a waveguide optic. Briefly, a 1 mm diameter biopsy punch was taken from both a Covid-19 and control sample, both of which were previously-scanned using a parallel beam. Sampleswere aligned *M* ≃ 40, resulting in an effective pixel size 152 µm. Images of the control sample were acquired by a sCMOS Camera (15µm Gadox scintillator, 2560 153 × 2160 pixel) with a physical pixel size of 6.5 µm (Andor Technology Ltd, UK). The Covid sample was recorded by a 1:1 fiber-coupled scintillator-based sCMOS camera (2048 x 2048 pixels, Photonic 155 Science, Sussex, UK) with a custom 15µm thick Gadox scintillator with pixel size of 6.5 µm. Photon energy was *E* = 10 keV for the control sample an *E* = 10.8 keV respectively. For the control sample, 1500 projections over 180 degrees were recorded with an acquisition time of 0.3 s, while for the Cov sample 1500 projections were acquired for four slightly different propagation distances The 3d structure of the cardiac tissue was reconstructed from the raw detector using the HOLOTOMOTOOLBOX (available at Lohse et al. 2020 (9)). Detailed paramters of the employed scanning procedure and reconstruction method are desribed in Reichart et al. 2021(10).

**Supplementary figure legends**

Supplementary figure 1: immunohistochemical viualisation of TIE-2 positive macrophages adherent to cardiac endothelial cells (A-D) and presence of ANGPT-2 on cardiac endothelial cells in COVID-19 (E-F).

A-D: Double immunohistochemistry of COVID-19 heart tissue for endothelial cells (CD31 (brown)) and TIE-2 positive macrophages (red) highlighting intraluminal and paravascular TIE-2 positive macrophages (arrowheads) directly adjacent to endothelial cells.

E-F: immunohistochemical staining for angiopoietin 2 (ANGPT-2) showing the presence of ANGPT-2 on endothelial cells in COVID-19 hearts (Panel E arrowheads), while no presence of ANGPT-2 could be detected in uninfected control hearts (Panel F, arrowheads).

G: Double immunohistochemistry of COVID-19 heart tissue for endothelial cells (CD31 (brown)) and TIE-2 (red) showing one of the few nicely preserved endothelial cell layers displaying a co-localisation of CD31- and TIE-2.

A-C: Magnification 400x, scale bar 20µm, D: magnification 600x, scale bar 10 µm, E-F: Magnification 200x, scale bar 20µm, G: Magnification 400x, scale bar 50µm

Supplementary figure 2: Additional scanning electron micrograph of microvascular corrosion casting depicting numerous blind-ending vessels with vanishing microvascular hierarchy and micro-extravasation (black arrowheads) indicative for microthrombi in COVID-19 heart tissue. The expansion of vascular plexus by intussusceptive pillars (black circle) is distinctly occurring in dilated vessel segments, preferably on sites of vessel branching. Scale bar 200 µm.

**Supplementary tables**

| Pat ID | Age | Gender | BMI | ECMO | Ventilation | Hospitalization (days) | cardiac involvement | Smoking | Hypertension | Diabetes Typ II | Immunosuppression | RAAS interacting drugs | catecholamin therapy | cardiac virus detection (PCR ct-value) | cause of death |
| --- | --- | --- | --- | --- | --- | --- | --- | --- | --- | --- | --- | --- | --- | --- | --- |
| 1 | 86 | M | 26 | No | No | 5 | No | No | Yes | Yes | Yes | Yes | No | Yes (15.5) | RF |
| 2 | 96 | M | 23 | No | No | 3 | No | No | Yes | No | No | Yes | No | Yes (20.2) | RF. Pneumonia |
| 3 | 78 | M | 44 | No | Yes | 3 | Yes | Yes | Yes | Yes | No | Yes | n.a. | Yes (24.2 | CRF |
| 4 | 66 | M | 29 | No | Yes | 9 | n.a. | Yes | Yes | No | No | Yes | n.a. | Yes (30.7) | RF |
| 5 | 74 | M | 27 | No | No | 3 | Yes | Yes | Yes | Yes | No | Yes | No | Yes (33.6) | RF. Pneumonia |
| 6 | 81 | F | 26 | No | No | 4 | No | Yes | Yes | No | No | Yes | No | Yes (33.7) | RF. Pneumonia |
| 7 | 71 | M | 25 | n.a. | n.a. | 0 | n.a. | No | No | No | No | No | n.a. | Yes (32.2) | RF. Pneumonia |
| 8 | 88 | M | 28 | n.a. | n.a. | 2 | n.a. | No | Yes | No | Yes | No | n.a. | Yes (32.3) | RF |
| 9 | 85 | M | 29 | n.a. | n.a. | 5 | Yes | Yes | Yes | No | No | Yes | n.a. | Yes (31.3) | RF into MOF |
| 10 | 58 | M | 47 | n.a. | n.a. | 7 | Yes | No | Yes | No | No | Yes | n.a. | Yes (33.1) | RF into MOF |
| 11 | 54 | M | 30 | n.a. | n.a. | 15 | Yes | No | No | No | No | No | n.a. | Yes (30.5) | RF |
| 12 | 72 | F | n.a. | No | Yes | 9 | No | n.a. | No | Yes | No | Yes | Yes | No | RF |
| 13 | 83 | F | n.a. | No | Yes | 12 | No | Yes | No | Yes | No | No | Yes | Yes (38.3) | MOF |
| 14 | 73 | M | 24.1 | No | Yes | 6 | Yes | n.a. | Yes | No | Yes | Yes | Yes | No | MOF. Rhabdomyolysis |
| 15 | 58 | M | 24.7 | Yes | Yes | 25 | Yes | No | Yes | Yes | No | No | Yes | No | ARDS. pneumonia |
| 16 | 77 | M | 31.6 | No | No | 2 | n.a. | No | Yes | Yes | No | Yes | No | No | ARDS. pneumonia |
| 17 | 79 | F | 36 | No | Yes | 14 | Yes | No | No | Yes | No | No | Yes | Yes (38.6) | ARDS into MOF |
| 18 | 74 | F | 26.46 | No | Yes | 5 | n.a. | n.a. | No | Yes | Yes | No | Yes | Yes (32.1) | RF |
| 19 | 66 | M | 37 | No | Yes | 27 | Yes | No | No | No | No | No | Yes | No | ARDS. Pneumonia |
| 20 | 74 | F | 27.7 | Yes | Yes | 14 | Yes | No | No | No | No | No | Yes | No | Enterothorax |
| 21 | 79 | F | 26.9 | Yes | Yes | 23 | n.a. | No | Yes | Yes | No | No | No | No | RF |
| 22 | 78 | F | 24.7 | Yes | Yes | 21 | n.a. | No | Yes | Yes | No | No | No | Yes (33.1) | RF |
| 23 | 62 | F | 26.3 | No | Yes | 62 | n.a. | No | Yes | No | Yes | Yes | Yes | Yes (39.7) | RF |
| 24 | 56 | M | 24.7 | Yes | Yes | 45 | Yes | No | No | No | No | No | Yes | No | RF |

Supplementary table 1A: COVID-19 patients characteristics

ARDS = Acute respiratory syndrome, BMI = body-mass-index, CRF = cardio-respiratory failure, ECMO = extracorporal membrane oxygenation, MOF = multi-organ-failure, n.a. = not available, RF = respiratory failure

| Pat ID | Gender | Age | BMI | ventilation | ECMO | Hospitalization (days) | cardiac involvement | smoking status | Hypertension | Diabetes Type II | Immunosuppression | RAAS interacting drugs | Catecholamine therapy | Subtype | cause of death |
| --- | --- | --- | --- | --- | --- | --- | --- | --- | --- | --- | --- | --- | --- | --- | --- |
| 1 | m | 74 | 41,4 | yes | Yes | 9 | Yes | Yes | Yes | No | No | Yes | Yes | H1N1 (2009) | CRF into MOF |
| 2 | m | 52 | 46,7 | yes | Yes | 4 | Yes | no | Yes | No | No | Yes | Yes | H1N1 (2009) | Myocarditis into MOF |
| 3 | w | 66 | 27 | yes | Yes | 17 | Yes | no | Yes | No | No | No | Yes | H1N1 (2009) | MOF |
| 4 | m | 53 | 35,2 | yes | Yes | 11 | Yes | Yes | Yes | No | No | No | Yes | H1N1 (2009) | CRF |
| 5 | m | 45 | 48,5 | yes | Yes | 8 | Yes | No | Yes | No | No | No | Yes | H1N1 (2009) | CRF |
| 6 | w | 59 | 28,6 | yes | Yes | 20 | Yes | No | No | No | No | No | Yes | H1N1 (2009) | RF |
| 7 | m | 56 | 36,7 | yes | Yes | 3 | Yes | No | No | No | No | No | Yes | H1N1 (2009) | CRF into MOF |
| 8 | m | 17 | 24,9 | yes | Yes | 41 | Yes | Yes | No | No | No | No | Yes | H1N1 (2009) | RF |
| 9 | m | 55 | 27,8 | yes | Yes | 24 | No | Yes | No | No | No | No | Yes | H1N1 (2009) | RF into MOF |
| 10 | w | 33 | 22 | yes | Yes | 1 | n.a. | No | No | No | No | No | Yes | H1N1 (2009) |  |
| 11 | m | 51 | 43,8 | yes | Yes | 13 | n.a. | No | No | No | No | No | Yes | H1N1 (2009) |  |
| 12 | w | 34 | 37,1 | yes | Yes | 16 | Yes | Yes | Yes | No | No | No | Yes | seasonal (B) |  |
| 13 | w | 75 | 19 | yes | No | 14 | n.a. | n.a. | No | No | No | No | Yes | seasonal (B) |  |
| 14 | w | 43 | n.a. | yes | No | 23 | n.a. | Yes | No | No | No | No | Yes | H1N1 (2009) |  |
| 15 | m | 60 | 37 | yes | No | 6 | No | Yes | Yes | No | No | No | Yes | H1N1 (2009) |  |
| 16 | w | 64 | 24,6 | yes | No | 16 | No | Yes | No | No | No | No | Yes | seasonal (A) |  |

Supplementary table 1B: Influenza patients characteristics

BMI = body-mass-index, CRF = cardio-respiratory failure, ECMO = extracorporal membrane oxygenation, MOF = multi-organ-failure, n.a. = not available, RF = respiratory failure

| Pat ID | Gender | Age | BMI | ventilation | ECMO | Hospitalization (days) | proven cardiac involvement | smoking status | Hypertension | Diabetes Type II | Immunosuppression | RAAS interacting drugs | catecholamine therapy | cause of death |
| --- | --- | --- | --- | --- | --- | --- | --- | --- | --- | --- | --- | --- | --- | --- |
| 1 | M | 57 | 24.4 | yes | No | n.a. | Yes | No | Yes | No | No | Yes | Yes | alive |
| 2 | M | 23 | 21.5 | no | No | n.a. | No | No | No | No | No | No | No | alive |
| 3 | M | 59 | 26.5 | n.a. | n.a. | n.a. | n.a. | Yes | Yes | Yes | No | Yes | n.a. | alive |
| 4 | M | 50 | 28.7 | yes | No | n.a. | Yes | Yes | No | Yes | No | No | Yes | alive |
| 5 | F | 25 | 22.4 | No | n.a. | n.a. | Yes | No | No | No | No | No | No | alive |
| 6 | M | 73 | n.a. | n.a. | n.a. | n.a. | n.a. | n.a. | n.a. | n.a. | n.a. | n.a. | n.a. | myocarditis |
| 7 | F | 71 | n.a. | n.a. | n.a. | n.a. | n.a. | n.a. | n.a. | n.a. | n.a. | n.a. | n.a. | myocarditis |
| 8 | F | 80 | n.a. | n.a. | n.a. | n.a. | n.a. | n.a. | n.a. | n.a. | n.a. | n.a. | n.a. | myocarditis |

Supplementary table 1C: conventional lymphocytic myocarditis patients characteristics

BMI = body-mass-index, ECMO = extracorporal membrane oxygenation, , n.a. = not available

| Pat ID | Gender | Age | BMI | smoking status | Hypertension | Diabetes Type II | Immunosuppression | RAAS interacting drugs | ECMO | surgery |
| --- | --- | --- | --- | --- | --- | --- | --- | --- | --- | --- |
| 1 | F | 41 | 26.7 | No | No | No | No | No | No | atrial myxoma |
| 2 | F | 26 | 22 | No | No | No | No | Yes | No | cardiac fibroma |
| 3 | F | 36 | 25.7 | No | No | No | No | No | No | valve replacement |
| 4 | F | 48 | 23.4 | No | No | No | No | No | No | valve replacement |
| 5 | F | 68 | 28.7 | No | Yes | No | No | Yes | No | valve replacement |
| 6 | M | 60 | 27.4 | Yes | Yes | No | No | Yes | No | Aorto-coronary-bypass |
| 7 | M | 65 | 30.7 | No | No | No | No | No | No | Aorto-coronary-bypass |
| 8 | F | 70 | 28.3 | No | Yes | No | No | Yes | No | valve replacement |
| 9 | F | 80 | 29 | No | Yes | Yes | No | Yes | No | valve replacement |

Supplementary table 1D: control patients characteristics

BMI = body-mass-index, ECMO = extracorporal membrane oxygenation, , n.a. = not available

| Pat ID | CRP | PCT (ng/L) | INR | D-Dimer (µg/mL) | LDH (U/l) | Kreatinin | Lactat (mmol/L) | pro-BNP (ng/L) | CK-MB (µg/l) | CK (U/L) | TroponinT (ng/L) | echocardiography |
| --- | --- | --- | --- | --- | --- | --- | --- | --- | --- | --- | --- | --- |
| COVID-19 | | | | | | | | | | | | |
| 1 | 218,4 mg/dL | n.a. | n.a. | n.a. | 321 | 120 µmol/l | n.a. | n.a. | n.a. | n.a. | n.a. | normal findings |
| 2 | 512,3 mg/dL | n.a. | n.a. | n.a. | 408 | 176 µmol/l | n.a. | n.a. | 4,7 | n.a. | n.a. | aortic valve stenosis (moderate severity) |
| 3 | 339,1 mg/dL | n.a. | 1,7 | n.a. | 1605 | 306 µmol/l | n.a. | n.a. | 10,3 | n.a. | n.a. | n.a. |
| 4 | 144,3 mg/dL | n.a. | 1,2 | n.a. | 236 | 800 µmol/l | n.a. | n.a. | 5,4 | n.a. | 36 | n.a. |
| 5 | 226,4 mg/dL | n.a. | 1,1 | 3,69 | 601 | 706 µmol/l | n.a. | n.a. | 1,7 | n.a. | 77 | n.a. |
| 6 | 82,1 mg/dL | n.a. | 1,1 | n.a. | 753 | 84 µmol/l | n.a. | n.a. | n.a. | n.a. | n.a. | n.a. |
| 7 | n.a. | n.a. | 2 | 1,17 | n.a. | 464 µmol/l | n.a. | n.a. | n.a. | n.a. | n.a. | n.a. |
| 8 | 113,3 mg/dL | n.a. | 1,6 | 1,13 | 290 | 65 µmol/l | n.a. | n.a. | n.a. | n.a. | 33 | LVEF 55%, moderate MI, dilated left atrium |
| 9 | 180 mg/dL | n.a. | 1,3 | 20 | 444 | 720 µmol/l | n.a. | n.a. | 3,3 | n.a. | 262 | n.a. |
| 10 | 13,6 mg/dL | n.a. | 3,7 | 5,48 | 5267 | 407 µmol/l | n.a. | n.a. | 53,6 | n.a. | 221 | n.a. |
| 11 | 172,7 mg/dL | n.a. | 1,4 | 6,93 | 1635 | 125 µmol/l | n.a. | n.a. | 31,2 | n.a. | 98 | severely reduced biventricular function (LVEF 10-20%) moderate MI |
| 12 | 175 mmol/L | n.a. | n.a. | n.a. | n.a. | n.a. | n.a. | n.a. | n.a. | n.a. | n.a. | n.a. |
| 13 | 57 mmol/L | n.a. | n.a. | n.a. | n.a. | n.a. | n.a. | n.a. | n.a. | n.a. | 44 | n.a. |
| 14 | 14 mg/dL | n.a. | n.a. | n.a. | n.a. | 1,51 mg/dL | 13,7 | 59275 | n.a. | 12370 | 14461,44 | moderate reduced LVEF, apical and anteroseptal akinesia |
| 15 | 200 mmol/L | n.a. | n.a. | n.a. | n.a. | n.a. | 14,9 | 28905 | n.a. | n.a. | 334 | normal LVEF, moderate AI, moderate PI |
| 16 | 96,6 mmol/L | n.a. | n.a. | n.a. | n.a. | n.a. | 1,5 | n.a. | n.a. | n.a. | n.a. | n.a. |
| 17 | 36 mg/dL | 42,2 | 1 | 10,06 | n.a. | 4,23 mg/dL | 15 | 2118 | n.a. | 3132 | n.a. | n.a. |
| 18 | 7,5 mg/dL | n.a. | n.a. | n.a. | n.a. | n.a. | 15 | 533 | n.a. | n.a. | 26,57 | n.a. |
| 19 | 335 mg/dL | n.a. | n.a. | n.a. | n.a. | n.a. | 10,5 | 34897 | n.a. | n.a. | 35 | moderate reduced LVEF, mild diffuse hypokinesia, mild AI, mild TI, mildly reduced RVEF |
| 20 | 79 mmol/L | n.a. | n.a. | n.a. | n.a. | n.a. | 1 | 1254 | n.a. | n.a. | 125 | normal LVEF, moderately reduced RVEF, moderate AI, moderate TI |
| 21 | n.a. | n.a. | n.a. | n.a. | n.a. | n.a. | n.a. | n.a. | n.a. | n.a. | n.a. | n.a. |
| 22 | n.a. | n.a. | n.a. | n.a. | n.a. | n.a. | n.a. | n.a. | n.a. | n.a. | n.a. | n.a. |
| 23 | 177 mmol/L | n.a. | n.a. | n.a. | n.a. | n.a. | 1,3 | n.a. | n.a. | n.a. | n.a. | minor MI, minor TI |
| 24 | 350 mmol/L | n.a. | n.a. | n.a. | n.a. | n.a. | 2,5 | 38260 | n.a. | n.a. | 117 | severely reduced RVEF, minor MI, minor TI |
| Influenza | | | | | | | | | | | | |
|  |  |  |  |  |  |  |  |  |  |  |  |  |
| 1 | 225 mg/L | 10,4 | n.a. | n.a. | 23315 | 78 | >15 | 5910 | 71 | 926 | 313 | minor AI, minor to moderate MI |
| 2 | 144 mg/L | 10,6 | 1,67 | n.a. | 3711 | 149 | 3,8 | 17488 | n.a. | 16989 | 198 | major reduction of LVEF |
| 3 | 386 mg/L | 2,2 | 4,35 | n.a. | n.a. | 64 | >15 | n.a. | 611 | 1746 | 56 | n.a. |
| 4 | 231 mg/L | 8,1 | 1,9 | n.a. | 3724 | 234 | 9,3 | 15007 | 789 | n.a. | <0,1 | moderate reduction of LVEF |
| 5 | 100 mg/L | 1,4 | 2,93 | n.a. | 13979 | 144 | >15 | 255 | 229 | 8415 | 0,18 | n.a. |
| 6 | 194 mg/L | 4,5 | 2,79 | n.a. | 11694 | 90 | 12,7 | 1418 | 378 | 1646 | n.a. | normal findings |
| 7 | 154 mg/L | 208,9 | 2,95 | 6,05 | 2322 | 272 | 12,5 | 16616 | 202 | 16061 | 0,26 | n.a. |
| 8 | 67 mg/L | 1,6 | 2,64 | 3,87 | 3059 | 30 | 1,5 | 475 | 45 | 2598 | <0,1 | mild to moderate reduced LVEF |
| 9 | 284 mg/L | 18,3 | 1,37 | n.a. | 434 | 150 | 10,1 | n.a. | n.a. | 30 | n.a. | normal findings |
| 10 | 230 nmol/L | n.a. | n.a. | n.a. | n.a. | n.a. | 1,7 | n.a. | n.a. | n.a. | n.a. | n.a. |
| 11 | n.a. | n.a. | n.a. | n.a. | n.a. | n.a. | 18 | n.a. | n.a. | n.a. | n.a. | n.a. |
| 12 | 230 nmol/L | n.a. | n.a. | n.a. | n.a. | n.a. | 11,3 | 294 | n.a. | n.a. | 10 | major reduction of RV-EF |
| 13 | 62,8 nmol/L | n.a. | n.a. | n.a. | n.a. | n.a. | 16 | 406,8 | n.a. | n.a. | n.a. | n.a. |
| 14 | 167,8 nmol/L | n.a. | n.a. | n.a. | n.a. | n.a. | 12,9 | 13034 | n.a. | n.a. | n.a. | n.a. |
| 15 | 194,3 nmol/L | n.a. | n.a. | n.a. | n.a. | n.a. | 14,6 | n.a. | n.a. | n.a. | n.a. | normal findings |
| 16 | 113,6 nmol/L | n.a. | n.a. | n.a. | n.a. | n.a. | 10 | n.a. | n.a. | n.a. | n.a. | normal findings |
| Myocarditis | | | | | | | | | | | | |
|  |  |  |  |  |  |  |  |  |  |  |  |  |
| 1 | 232 | 5,5 | 1,74 | 2,04 | 1327 | 208 | 2,04 | 9555 | 181 | 1025 | n.a. | severely reduced LVEF (15%) and severely reduced RVEF (10-15%) |
| 2 | 3 | <0,1 | 2,3 | n.a. | 238 | 84 | 1,27 | 163 | 18 | 88 | n.a. | severely reduced LVEF (20-25%) |
| 3 | n.a. | n.a. | n.a. | n.a. | n.a. | n.a. | n.a. | n.a. | n.a. | n.a. | n.a. | n.a. |
| 4 | 67 | 2,3 | 2,09 | n.a. | 623 | 266 | 3,25 | >35000 | 112 | 1469 | n.a. | severely reduced LVEF (15%) and RVEF, dilatation left atrium |
| 5 | 29 | 0,5 | 1,3 | n.a. | 468 | 163 | 2,1 | >35000 | 31 | 180 | 1225 | severely reduced LVEF (25%) |
| 6 | n.a. | n.a. | n.a. | n.a. | n.a. | n.a. | n.a. | n.a. | n.a. | n.a. | n.a. | n.a. |
| 7 | n.a. | n.a. | n.a. | n.a. | n.a. | n.a. | n.a. | n.a. | n.a. | n.a. | n.a. | n.a. |
| 8 | n.a. | n.a. | n.a. | n.a. | n.a. | n.a. | n.a. | n.a. | n.a. | n.a. | n.a. | n.a. |

Supplementary table 2: laboratory and echocardiographic findings: AI = aortic valve insufficiency; CK = creatine kinase; CK-MB = creatine kinase MB-isoform; CRP = c-rreactive protein; INR = Prothrombin time (International normalized ratio); LVEF = left ventricule ejection fraction; MI = mitral valve insufficiency; PCT = procalcitonine; pro-BNP = pro- brain natriuretic peptide; RVEF = right ventricle ejection fraction; TI = tricuspidal insufficiency

|  | CD4 | CD8 | CD20 | CD68 | CD11b | TIE2 | CD16+ CD163- | CD16+ CD163+ | CD16+, CD163+, S100A9+ |
| --- | --- | --- | --- | --- | --- | --- | --- | --- | --- |
| COVID-19 | 4.09  ± 0.04 | 1.86  ± 0.03 | 0.40  ± 0.04 | 65.16 ±0.79 | 11.51 ±14.32 | 0.83 ±0.91 | 234.58 ±340.96 | 141.43 ±129.28 | 42.43 ±34.37 |
| Influenza | 4.16  ± 0.04 | 3.98  ± 0.04 | 2.154  ± 0.02 | 130.21 ±1.04 | 21.42 ±20.04 | 0.22 ±0.42 | 202.267 ±256.52 | 32.6 ±34.59 | 68.31 ±45.03 |
| Myocarditis | 32.06 ±0.74 | 47.10 ±0.76 | 13.44 ±0.03 | 72.52 ±1.59 | 78.03 ±110.08 | 1.48 ±2.36 | 371 ±382.51 | 1112.75 ±1683.42 | 84.13 ±59.66 |
| Control | 11.63  ± 0.19 | 11.20  ± 0.16 | 0.29  ± 0.02 | 12.49  ± 0.30 | 4.96 ±7.40 | 0.27 ±0.20 | n.a. | n.a. | n.a. |

Supplementary table 3: Inflammatory cell infiltrate characterization in COVID-19, Influenza and lymphocytic myocarditis. Values given are mean cells per mm² tissue ± SD.

**Supplementary References**

1. Akoya Biosciences I Opal Multiplex IHC Assay Development Guide and Image Acquisition Information. https://www.akoyabio.com/wp-content/uploads/2020/03/Akoya_Opal_Assay_Development_Guide.pdf

2. von Stillfried S, Villwock S, Bülow RD, et al (2021) SARS-CoV-2 RNA screening in routine pathology specimens. Microb Biotechnol 14:1627–1641. https://doi.org/10.1111/1751-7915.13828

3. Remmelink M, De Mendonça R, D’Haene N, et al (2020) Unspecific post-mortem findings despite multiorgan viral spread in COVID-19 patients. Crit Care 24:1–10. https://doi.org/10.1186/s13054-020-03218-5

4. Ackermann M, Stark H, Neubert L, et al (2020) Morphomolecular motifs of pulmonary neoangiogenesis in interstitial lung diseases. Eur Respir J 55:. https://doi.org/10.1183/13993003.00933-2019

5. Neubert L, Borchert P, Stark H, et al (2020) Molecular Profiling of Vascular Remodeling in Chronic Pulmonary Disease. Am J Pathol. https://doi.org/10.1016/j.ajpath.2020.03.008

6. Ashburner M, Ball CA, Blake JA, et al (2000) Gene ontology: Tool for the unification of biology. Nat Genet 25:25–29. https://doi.org/10.1038/75556

7. Carbon S, Douglass E, Good BM, et al (2021) The Gene Ontology resource: Enriching a GOld mine. Nucleic Acids Res 49:D325–D334. https://doi.org/10.1093/nar/gkaa1113

8. Mi H, Muruganujan A, Ebert D, et al (2019) PANTHER version 14: More genomes, a new PANTHER GO-slim and improvements in enrichment analysis tools. Nucleic Acids Res 47:D419–D426. https://doi.org/10.1093/nar/gky1038

9. Lohse LM, Robisch AL, Töpperwien M, et al (2020) A phase-retrieval toolbox for X-ray holography and tomography. J Synchrotron Radiat 27:852–859. https://doi.org/10.1107/S1600577520002398

10. Reichardt M, Jensen PM, Dahl VA, et al (2021) 3D virtual Histopathology of Cardiac Tissue from Covid-19 Patients based on Phase-Contrast X-ray Tomography. Elife 10:1–28. https://doi.org/10.7554/eLife.71359
