# Supplementary figures and images for: Inflammation and vascular remodeling in COVID-19 hearts
Source: Angiogenesis. 2022 Nov 12;26(2):233–48. doi: 10.1007/s10456-022-09860-7 (PMC9660162; doi:10.1007/s10456-022-09860-7)

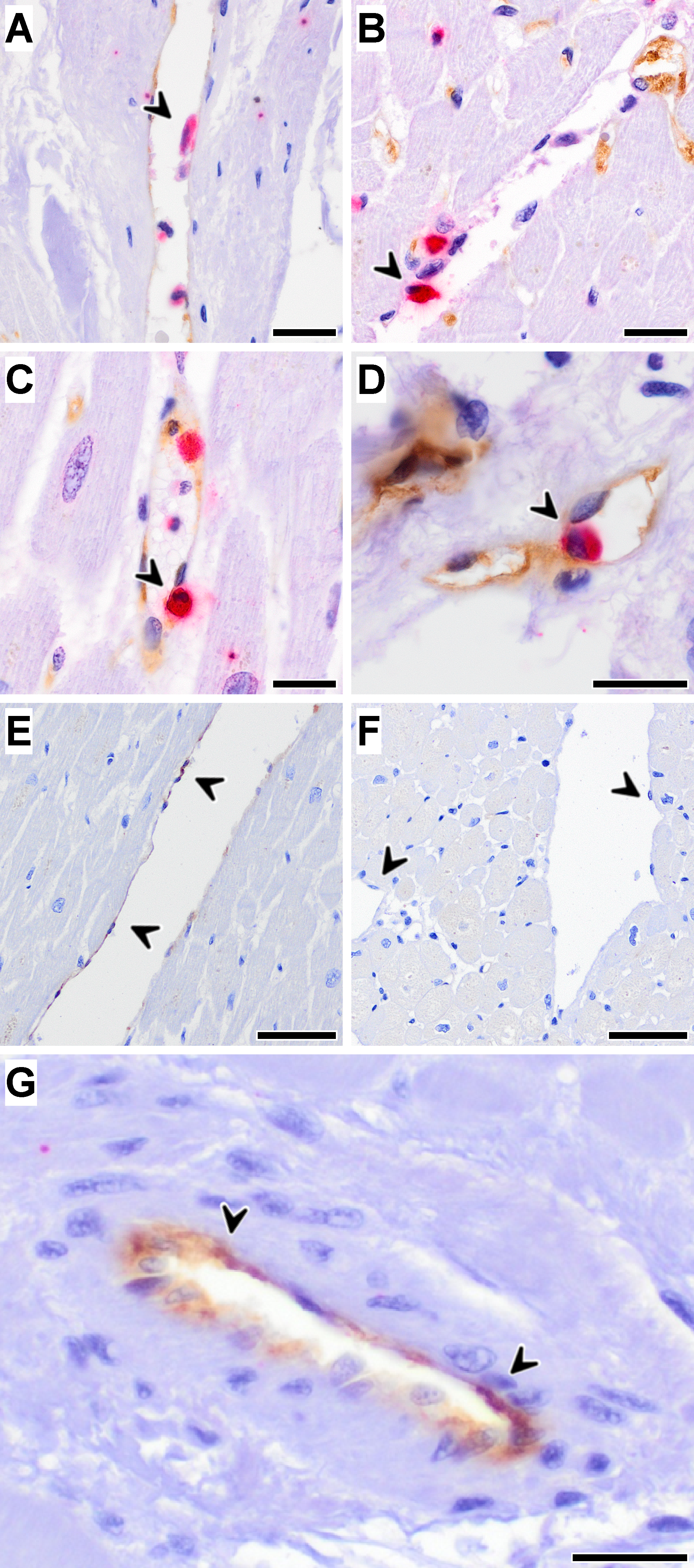

Supplement: Supplementary file 5 — Supplementary file5 (TIF 14928 kb) [file 10456_2022_9860_MOESM5_ESM.tif]
